# Recent Progress, Challenges, and Future Perspectives of Phase Change Materials for Building Thermal Energy Storage

**DOI:** 10.3390/ma19214435

**Published:** 2026-10-22

**Authors:** Yunzhang Li, Simeng Li, Zhenglin Han, Tao Ding

**Affiliations:** 1 Taizhou Key Laboratory of Intelligent Construction and Low-Carbon Technology, School of Civil Engineering and Architecture, Taizhou Vocational and Technical College, Taizhou 318000, China; 2 State Key Laboratory Advanced Special Steel, Shanghai Applied Radiation Institute, Shanghai University, Shanghai 200444, China

**Keywords:** phase change materials, latent-heat thermal energy storage, building envelope, shape-stabilized composites, microencapsulation, thermal comfort, demand response, building energy efficiency

## Abstract

Integrating phase change materials (PCMs) into building fabrics and thermal systems enables these components to store and release latent heat. This article presents a structured critical narrative review of 120 peer-reviewed sources published from 2004 to 2026, systematically retrieved from major academic databases. Evidence across experimental, numerical, and field studies was classified by scale and evaluated against material, system, economic, and environmental metrics. Crucially, our synthesis reveals that nominal enthalpy alone poorly predicts actual building value. At the material level, representative composites exhibit nominal enthalpies of 43.31–229.7 J g^−1^, yet usable capacity is often reduced by inactive supports, incomplete transitions, and heat-transfer limitations. Conductive additives can enhance thermal conductivity by up to 156.4%, but also displace active PCM and increase viscosity, cost, and embodied impacts. At building scale, concrete-based components yield modest peak-temperature reductions (1.0–1.81 °C), whereas a full-scale lightweight envelope achieved 5–8 °C attenuation in favorable seasons; however, PCM was active for only 20–28% of monitored time and remained melted during summer. An active 7000 L salt-hydrate installation delivered only 36% of nominal capacity, while a numerical heat-pump/PCM study reported up to 42% operating-cost reduction. These findings show that activated fraction, regeneration, and system boundary are more informative than nominal enthalpy alone. To bridge this gap, we propose an application-first pathway linking service conditions, containment, and heat-transfer design. Future priorities include standardized multiscale testing, multi-season matched field trials, transparent capacity definitions, and explicit life-cycle assessments.

## 1. Introduction

Buildings experience a persistent temporal mismatch between heat gains or losses and the availability, cost, and carbon intensity of energy. Conventional insulation suppresses heat transmission but cannot deliberately absorb a daytime surplus and release it at a later hour. Thermal energy storage (TES) introduces precisely this time dimension. Among sensible, latent, and thermochemical routes, latent-heat TES based on phase change materials (PCMs) is particularly attractive for buildings, since a substantial quantity of heat can be stored across a narrow temperature interval within a comparatively small volume. The concept has advanced from early energy-conservation proposals to component-scale products and actively controlled storage systems [1,2,3].

A PCM stores sensible heat before and after transition and latent heat during transition. For a mass m heated from T_1_ to T_2_ through a melting interval T_m_, the ideal stored energy is expressed as:

$$Q=m[\int_{T1}^{Tm}Cp,s(T)dT+\Delta Hpc+\int_{Tm}^{T2}Cp,l(T)dT]$$

where m is the PCM mass, Cp,s and Cp,l are the solid- and liquid-state heat capacities and ΔHpc is the phase-change enthalpy. While this relationship emphasizes the advantage of a high phase-change enthalpy, it highlights a foundational engineering constraint: latent capacity is activated only when ambient thermal boundary conditions drive the material across its transition window. A high-enthalpy PCM that remains persistently solid or liquid functions purely as sensible thermal mass, potentially compromising energy efficiency by adding dead weight or undesirable thermal resistance. Consequently, material selection requires dynamic alignment with localized climatic conditions, envelope placement, HVAC operating parameters, occupancy profiles, and diurnal charging/discharging schedules [4,5,6]. The ideal expression assumes a specified initial and final state, a complete phase transition, negligible heat loss, and equilibrium properties. When melting or freezing occurs over a distributed interval, the enthalpy–temperature relation, or an experimentally derived apparent heat capacity, should be integrated instead of assigning all latent heat to one melting temperature.

The research field has expanded along two coupled directions. Materials research has sought higher storage density, lower supercooling, faster heat transfer, reduced leakage, improved fire performance, and renewable or waste-derived constituents. Building research has examined incorporation in gypsum, mortar, concrete, brick, timber, windows, roofs, air-handling systems, cold-storage units, heat pumps, and solar-assisted systems. Recent reviews provide broad accounts of passive applications, cooling and heating systems, inorganic PCMs, and envelope integration [7,8,9,10,11,12,13]. Nevertheless, a gap remains between material-centered studies that report differential scanning calorimetry (DSC) results and building-centered studies that need usable capacity, heat-flux reduction, comfort, load shifting, durability, and cost.

This review addresses that gap through a multiscale structure–property–performance perspective, organized around four questions: (i) which material and composite advances resolve application-relevant failure modes; (ii) how incorporation route, location, and control determine storage utilization; (iii) which characterization and modeling practices support reliable translation from milligrams to buildings; and (iv) what evidence remains outstanding for safe, durable, and financeable deployment. The intended contribution is not a further catalog of PCMs but an application-first synthesis linking molecular and pore-scale confinement to component behavior, whole-building operation, and life-cycle value.

The intended audience includes materials scientists developing PCMs and supports; civil, architectural, and mechanical engineers integrating storage into assemblies and HVAC systems; building-energy modelers and control researchers; product manufacturers; and decision makers who require traceable evidence on durability, safety, cost, and environmental performance.

Table 1 shows that previous reviews have developed valuable but often separate strands of evidence: broad building applications and materials, heating and hybrid systems, passive envelopes, cooling, or inorganic PCMs. Taken together, they establish the breadth of the field but do not consistently connect material selection and containment to activated capacity, measured component/building outcomes, control conditions, and service-based economic and environmental boundaries. The present review synthesizes these strands through a common multiscale evidence framework and an application-first decision pathway, while distinguishing laboratory, component, numerical, and full-scale/field findings.

## 2. Review Methodology and Analytical Framework

Methodologically, this work is designed as a structured critical narrative review rather than a strict systematic review or meta-analysis. Relevant literature published between 2004 and 2026 was retrieved from major academic databases, including the Web of Science Core Collection, Scopus, ScienceDirect, and Google Scholar. The core search string was (“phase change material*” OR “PCM”) AND (building* OR envelope OR wall OR roof OR floor OR HVAC OR “heat pump”) AND (“thermal energy storage” OR “latent heat”). This search was supplemented with topic-specific terms related to encapsulation, shape stabilization, salt hydrates, bio-based materials, durability, fire safety, modeling, control strategies, economic performance, and life-cycle assessment.

The journal articles and reviews were eligible when they addressed building thermal storage or provided directly transferable material characterization, heat-exchanger, control, durability, safety, economic, or environmental evidence. Experimental, numerical, full-scale/field, and review studies were retained. Duplicate records, inaccessible full texts, studies without a building or transferable thermal-storage connection, and records that did not provide interpretable methods or outcomes were excluded. The retained corpus comprised 120 unique references after duplicate and eligibility screening; no initial hit count is reported because searches were iteratively expanded for citation chaining.

For each quantitative study, extraction fields included PCM family; transition interval; enthalpy basis; density, conductivity, supercooling, and PCM fraction when reported; containment and geometry; loading and location; climate and boundary conditions; baseline and control strategy; test or simulation duration; durability and safety protocol; and economic or LCA boundary. Evidence is labeled as laboratory, component, numerical, full-scale/field, or review evidence. “N.R.” denotes a variable not reported in the source. A statistical meta-analysis was not attempted because formulations, normalization bases, climates, boundary conditions, and performance endpoints were too heterogeneous for defensible pooling.

## 3. Fundamentals and Application-Oriented Selection

### 3.1. Thermal, Physical, and Safety Requirements

For passive indoor temperature regulation, the melting interval is conventionally positioned near the desired comfort band; for cold storage it must correspond to the supply and return temperatures of the cooling loop; and for heating storage it must remain compatible with heat-pump or solar-source temperatures. A narrow transition delivers strong temperature clamping yet may remain inactive under variable seasonal conditions, whereas a broader, cascaded, or tunable transition enlarges annual opportunity at the expense of isothermal storage intensity. Melting and freezing curves both require reporting, since supercooling and hysteresis jointly determine whether stored heat is released at a useful temperature.

High latent heat per unit mass proves insufficient once density, inactive support, shell fraction, or incomplete transition erodes volumetric and usable capacity. Thermal conductivity is similarly contextual. Low conductivity preserves a temperature gradient in passive boards yet constrains charging power in compact active units. Conductive fillers, fins, and metal foams shorten melting time while occupying volume, adding embodied impact and cost, and diminishing latent heat per unit composite mass. Optimization must accordingly target system-level power and capacity in combination, as nanoparticle- and metal-foam studies demonstrate [14,15].

Containment is mandatory for solid–liquid PCMs. Leakage stains finishes, alters thermal contact, generates odors or emissions, and compromises fire resistance, while volume change imposes stress on shells and pores. For inorganic salt hydrates, phase segregation, dehydration, corrosion, and supercooling constitute the primary concerns; for many organic PCMs, low conductivity and flammability dominate. Compatibility with binders is equally consequential, as alkaline pore solution, mixing shear, curing heat, moisture transport, and repeated phase-volume change may damage capsules or perturb hydration. Mechanical strength, water absorption, vapor permeability, freeze–thaw resistance, and reaction-to-fire performance therefore warrant evaluation alongside thermal storage [10,13].

### 3.2. PCM Families and Classification

Paraffins offer reproducible melting, chemical inertness, limited supercooling, and a broad commercial temperature range, offset by low conductivity, flammability, fossil origin, and leakage in the molten state. Fatty acids and their esters exhibit congruent melting and admit renewable feedstocks, while eutectic blending permits adjustment of the transition temperature [16]. Polyethylene glycol (PEG) is attractive because molecular weight governs its transition and because it interacts favorably with numerous porous matrices; matrix chemistry and retained PEG fraction jointly determine the effective enthalpy [17].

Salt hydrates offer high volumetric storage density, relatively high conductivity, non-flammability, and low raw-material cost. They may nevertheless supercool, segregate during incongruent melting, lose water, and corrode metals. Stabilization combines nucleating agents, thickening or gelling networks, porous supports, and moisture-resistant coatings. A polyurethane-acrylate-coated salt-hydrate/diatomite composite, for example, combined a reported latent heat of 102.6 J g^−1^ with only 0.5 °C supercooling and suppressed dehydration [18]. Hydrogel confinement and engineered eutectic hydrates are more recent routes toward form stability and repeatable crystallization [19,20].

Bio-based PCMs include vegetable fats and oils, beeswax, bio-derived fatty acids or esters, and biomass-supported composites. They can reduce fossil dependence and create circular value, but the label ‘bio-based’ does not guarantee low environmental impact or stable composition. Oxidation, odor, feedstock variability, land-use implications, and fire behavior require evaluation. Vegetable fat has been demonstrated as a low-cost building PCM, and broader reviews identify viable temperature ranges among vegetable-oil derivatives [21,22,23]. Beeswax has also been combined with graphene and wood scaffolds to improve transport and containment [24,25].

Eutectic mixtures are valuable when no single material provides the required transition temperature. Organic–organic systems can retain low supercooling, while salt-hydrate eutectics can increase volumetric capacity. Composition control is critical because impurities and cycling-induced separation may shift the phase diagram. Cascaded systems deliberately use multiple PCMs at different temperatures; tunable concepts adjust the transition in situ. Modeling indicates that well-designed cascaded or tunable envelopes can remain active over more of the year than a single fixed-temperature layer [26].

This multi-axis taxonomy avoids treating chemistry, origin, and product form as interchangeable categories. Across these families, suitability is determined not by the label alone but by transition-temperature matching, usable volumetric capacity, containment, compatibility, safety, and the ability to regenerate under the intended building schedule [10,12].

## 4. Composite Engineering and Recent Material Progress

### 4.1. Encapsulation and Shape Stabilization

Table 2 collates representative PCM and composite formulations across organic, inorganic, eutectic, bio-based, cementitious, and geopolymer systems. For each study, it reports transition temperature, enthalpy basis, conductivity, PCM loading, cycling evidence, and the principal limitation when these data are available; N.R. denotes information not reported. Concrete and geopolymer examples connect material-level properties with construction-relevant performance.

Macroencapsulation locates PCM within panels, pouches, tubes, spheres, or channels, affording a high PCM fraction and replaceability while demanding careful sealing, accommodation of volume change, and sufficient heat-transfer area. Microencapsulation surrounds droplets with polymeric or inorganic shells and permits direct mixing into plaster or mortar, improving dispersion and leakage control; shell mass nevertheless depresses effective enthalpy, capsules rupture under mixing shear, and the soft inclusions introduce porosity and reduce strength. Agglomerated microcapsules with thickened shells have been proposed to withstand mortar processing, although increased dosage still degraded flow and mechanical performance [36,37]. Multi-temperature microcapsules can broaden operational coverage [38].

Shape-stabilized PCMs immobilize the liquid phase through capillarity, adsorption, or a polymer network. Porous minerals including diatomite, kaolinite, montmorillonite, palygorskite, zeolite, expanded perlite, silica, and titanium dioxide are inexpensive, non-combustible, and frequently compatible with construction binders. For this review, leakage-onset temperature means the lowest temperature at which visible liquid exudation is first detected during a controlled heating test; it is not a time-to-leakage value and is protocol dependent. Vacuum impregnation can increase pore filling, but excessive PCM blocks transport pathways and weakens the supporting skeleton. Examples include lauryl alcohol/kaolinite, methyl palmitate/palygorskite, stearic acid/TiO_2_, LA–PA/nano-SiO_2_, and capric–stearic acid/montmorillonite composites [28,29,39,40,41,42,43].

Carbonaceous supports and fillers—expanded graphite, graphene, carbon nanotubes, carbonized biomass, and carbon aerogels—establish continuous conductive pathways at low loading. In a capric–stearic acid/montmorillonite composite, 2 wt.% graphene raised thermal conductivity by 156.4% while preserving cycling stability [28], and 0.3 wt.% graphene in beeswax was reported to increase latent heat and produce a conductivity of 2.8 W m^−1^ K^−1^ [25]. These gains are formulation-specific and should not be generalized without considering dispersion, anisotropy, percolation, electrical conductivity, embodied impacts, and reduced PCM fraction.

Let $w_{PCM}$ denote the mass fraction of active PCM in a composite and ${\Delta H}_{neat}$ the measured phase-change enthalpy of the neat PCM. The dilution-only enthalpy expected from PCM mass fraction alone is:

$${\Delta H}_{dilution}=w_{PCM}\times {\Delta H}_{neat}$$

The ratio of measured composite enthalpy to this dilution-only expectation is:

$$R_{H}=\frac{{\Delta H}_{composite}}{{\Delta H}_{dilution}}$$

Values below unity indicate additional crystallization or phase-transition loss beyond simple support/shell dilution; they should not automatically be attributed to dilution alone.

Biogenic and waste-derived scaffolds constitute a prominent direction. Carbonized maize straw, bamboo, wood templates, textile waste, seashell powder, phosphogypsum, Mardin stone, and industrial by-products couple pore confinement with resource valorization [30,33,44,45,46,47,48,49]. Hierarchical wood and bamboo architectures merit particular attention, since aligned channels establish anisotropic heat-transfer pathways while surface modification governs wettability and retention. The strongest sustainability claims nonetheless demand functional-unit life-cycle assessment, because drying, carbonization, purification, vacuum processing, and conductive additives may offset feedstock advantages.

Three converging composite-design routes emerge from the broader evidence. Encapsulation methodology increasingly couples shell selection to operating temperature and manufacturing constraints, while mineral, three-dimensional carbon, graphene-aerogel, and nanotube scaffolds are engineered as connected transport and containment networks [50,51,52,53,54,55,56]. In parallel, biomimetic, flax-fiber, and other bio-derived supports expand the renewable design space, but require explicit control of feedstock variability, interfacial stability, and end-of-life recovery [57,58,59]. Recent model-driven and low-cost stabilization studies also shift attention from isolated peak properties toward durable, scalable formulations [60,61].

**Figure 1:**
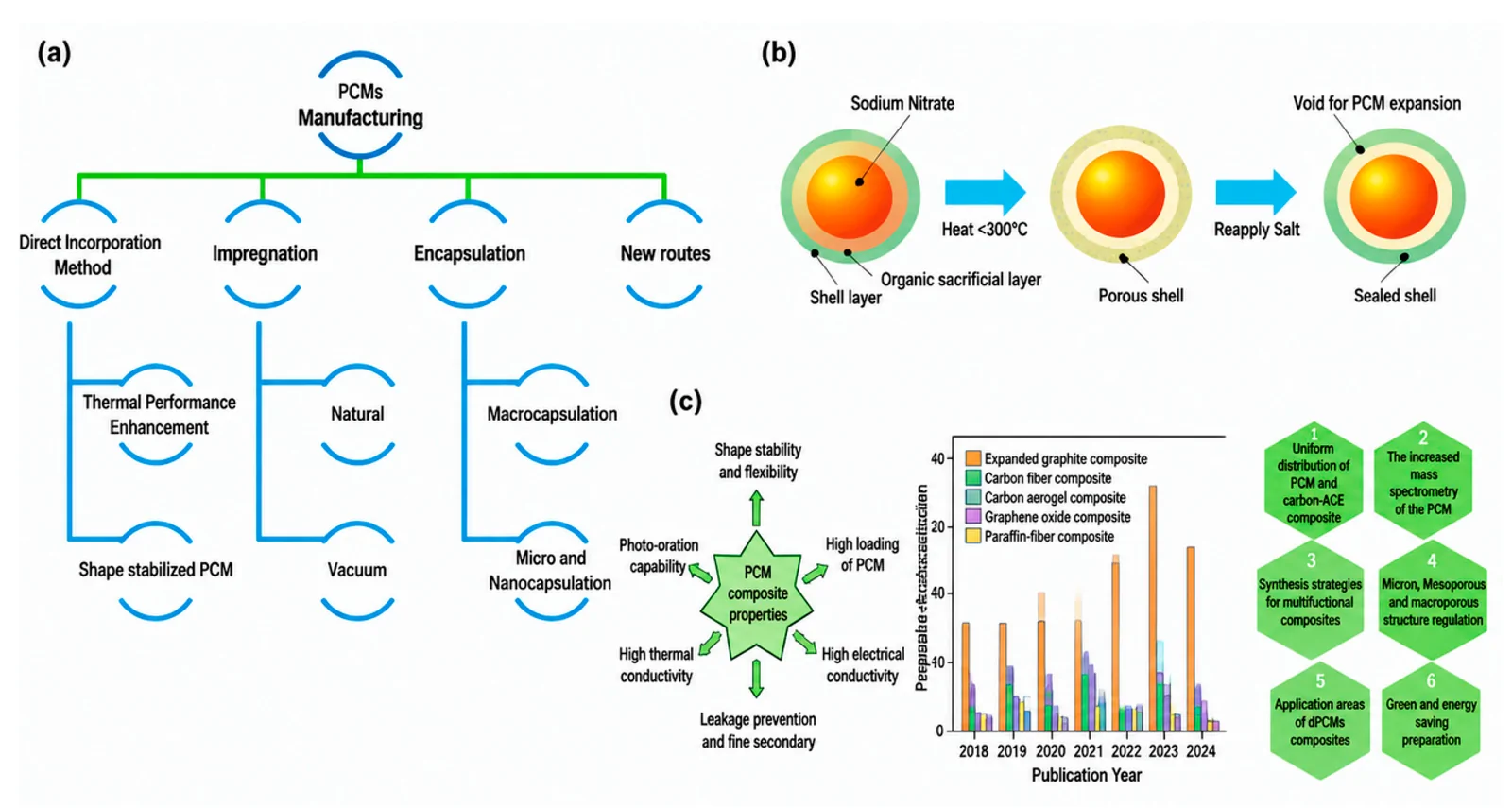
Representative containment and composite-development routes for PCMs: (**a**) manufacturing routes from direct incorporation to impregnation and encapsulation; (**b**) schematic of the investigated sodium nitrate encapsulated capsules [51]; and (**c**) functions and research directions of three-dimensional carbon/PCM composites [50].

Figure 1a maps principal integration and containment routes; Figure 1b shows the sodium-nitrate capsule architecture, including the shell and expansion void; and Figure 1c summarizes reported functions and design priorities for three-dimensional carbon/PCM composites. The bar graph in panel (c) provides publication-year context for reported composite-property improvements rather than a cross-study performance metric used in this review. Together, the panels support an application-first choice of containment: direct incorporation and porous impregnation favor process simplicity, whereas encapsulation accommodates liquid-phase change and carbon frameworks can combine retention with heat-transfer pathways. These benefits remain subject to inactive mass, cost, fire, and electrical constraints [50,51].

### 4.2. Multifunctional Enhancement

Thermal-conductivity enhancement is beneficial only when conduction is the dominant limitation in the charge–discharge process. Nanoparticles can increase effective conductivity, but they may agglomerate, sediment, alter viscosity, suppress natural convection, or reduce latent heat per unit mass. Expanded graphite, carbon frameworks, metal foams, fins, and compact heat exchangers generally provide more continuous heat-transfer pathways. Their use must nevertheless be evaluated against inactive mass, fabrication complexity, pressure drop, corrosion, recyclability, and cost [15,62,63].

The reported graphene cases illustrate both promise and caution. A 2 wt.% graphene addition produced a 156.4% conductivity increase, whereas 0.3 wt.% graphene in beeswax reached approximately 2.89 W m^−1^ K^−1^ and was accompanied by a reported latent-heat increase. These outcomes are formulation-specific: filler loading, orientation, contact resistance, anisotropy, viscosity, and displaced PCM mass must be reported. The proposed Brownian-motion and particle-clustering explanation for the beeswax result remains a study-level interpretation rather than a generally established mechanism [25,28].

Flame safety is a decisive barrier for organic PCMs in occupied buildings. Inorganic shells and supports, zinc borate, mineral binders, intumescent additives, and compartmentalized macrocontainers can reduce ignition and flame spread. The n-hexadecane/zinc-borate composite illustrates a flame-retardant formulation, whereas polyethylene/paraffin composites illustrate polymer-network containment and leakage control [64,65]. Nevertheless, thermogravimetric stability or a small-flame observation is not equivalent to a building-product fire classification. Heat release rate, smoke production, toxicity, dripping, and the response of the complete assembly must be tested after aging.

The mechanical penalty of PCM addition is equally important in cementitious products. Soft capsules, weak porous aggregates, and additional interfacial transition zones commonly lower compressive and flexural strength. The appropriate comparison is application-dependent: a non-structural interior board does not need structural-concrete strength, but it must survive handling, fasteners, impact, moisture, and fire. Matrix refinement using supplementary binders, geopolymer networks, coatings, or pre-placed impregnated aggregates can partly decouple storage from load-bearing performance [34,35,66,67,68,69,70].

New cement-based studies further show that form-stable PEG and paraffinic or bio-based microcapsules can be tailored for mortar integration, including reduced-graphene-oxide enhancement, but the thermal gain remains coupled to workability, strength, and dispersion quality [71,72].

**Figure 2:**
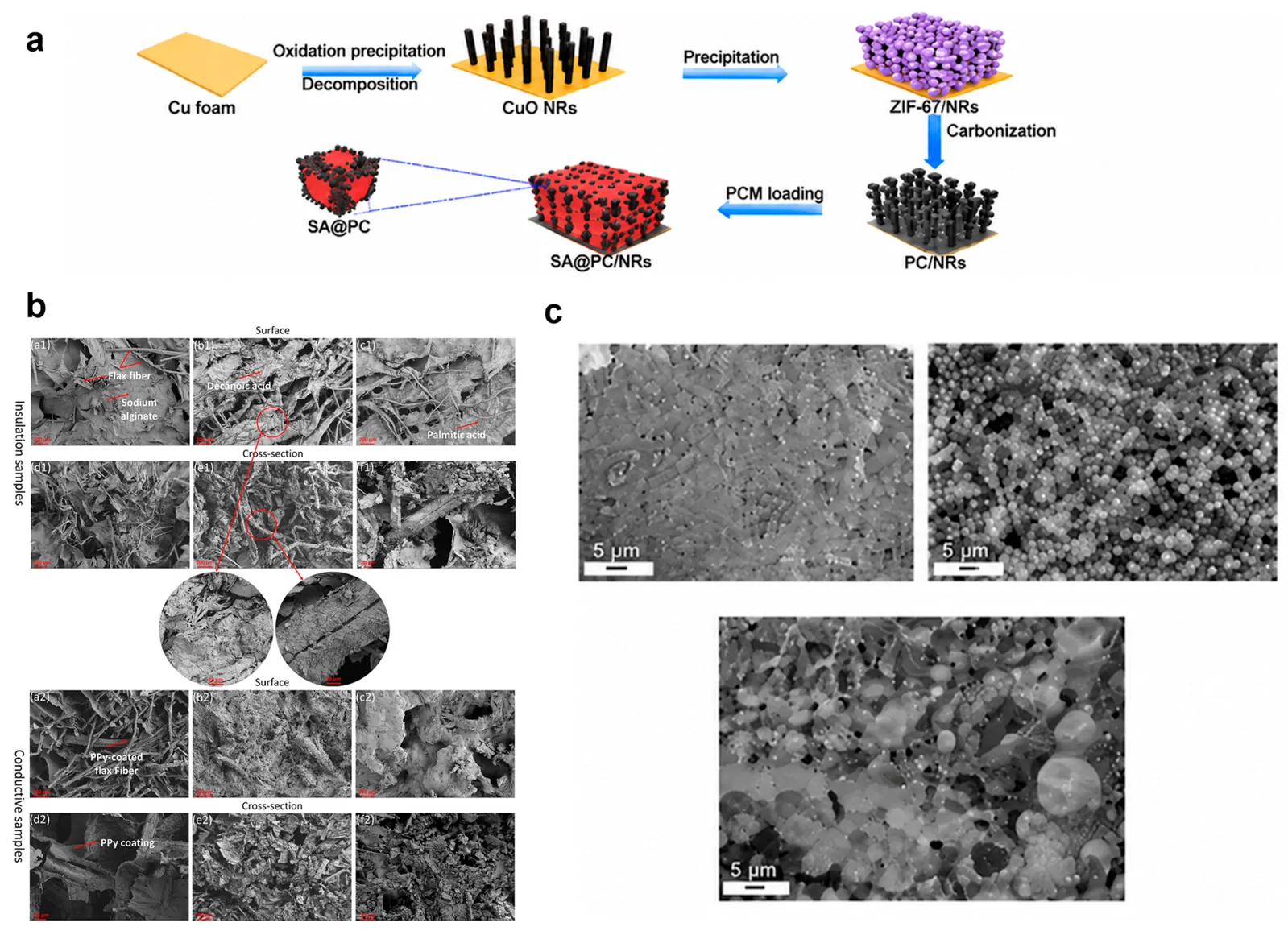
Representative support architectures and composite morphologies: (**a**) fabrication of a hierarchical carbon scaffold for shape-stabilized PCM loading [50]; (**b**) flax-fiber and polypyrrole-modified bio-based PCM microstructures [59]; and (**c**) microstructures of cement-based materials containing paraffinic or bio-based microencapsulated PCMs [71].

Figure 2a is a fabrication schematic, whereas panels (b,c) are microscopy images. The SEM panels reveal scaffold topology, pore structure, capsule dispersion, and interfacial contact, but they do not by themselves prove heat-transfer pathways or leakage resistance. The conceptual sequence is PCM loading → pore filling/capillary retention → a connected scaffold that may shorten heat-conduction paths; each functional effect requires independent calorimetric, leakage, and thermal-response evidence. These observations explain why the surrounding matrix cannot be treated as inert and why composite-scale performance depends on morphology and interfaces [50,59,71].

### 4.3. Reliability and Compatibility

Cycling reliability is commonly asserted on the basis of 100–1000 DSC or oven cycles. Such tests serve as useful screens yet fail to reproduce daily and seasonal dwell times, incomplete transitions, humidity, UV exposure, freeze–thaw, salt crystallization, alkaline attack, or mechanical restraint. Mass loss, transition drift, enthalpy retention, shell integrity, leakage, corrosion, and mechanical properties warrant concurrent measurement. Multiscale characterization linking molecular chemistry, pore-scale distribution, component heat flow, and accelerated aging offers a substantially stronger qualification route [73]. Heritage lime mortars impose the additional requirements of compatibility, reversibility, vapor transport, and benign substrate interaction, which remain under-tested [74].

## 5. Integration into Building Components

### 5.1. Gypsum, Mortar, Concrete, and Masonry

Table 3 compares representative passive-component and construction-material studies across laboratory, numerical, and full-scale/field evidence. It distinguishes material-level feasibility from component/building outcomes; detailed concrete and geopolymer properties are summarized in Table 2. The salt-hydrate example is the paired full-scale light-steel-frame study [75], whose seasonal temperature attenuation and limited activation are reported separately from laboratory material data.

Gypsum wallboard and plaster are favorable hosts because they are interior-facing, lightweight, inexpensive, and exposed to the indoor thermal cycle. PCM may be dispersed as microcapsules, impregnated in porous inclusions, or contained in tubes. A gypsum panel containing paraffin in copper tubes demonstrated leakage control and enhanced storage, while a gypsum/C18–C24 composite reported a melting temperature of 26.74 °C and latent heat of 43.31 J g^−1^ with stable behavior after 1000 cycles [27,77]. Integrating lauric acid/zeolite/graphite into gypsum combines containment and conductivity enhancement [78]. The decisive component metric is not composite enthalpy alone but heat-flux phase shift and the fraction of material that cycles under realistic surface temperatures.

Mortar and concrete offer large available mass and direct integration into construction, but structural and thermal functions compete. Direct impregnation can interfere with hydration and leak; macroencapsulated aggregates protect PCM but introduce weak inclusions; microcapsules disperse storage but are vulnerable to mixing shear. Alkali-activated and geopolymer binders may accommodate recycled supports and reduce clinker-related impacts [34,35,67]. PCM concrete panels, Mardin-stone composites, phosphogypsum paints, air-lime mortars, and self-leveling mortars illustrate the widening range of products [32,33,49,66,70]. Each route needs a declared functional role and corresponding mechanical, hygrothermal, and fire acceptance criteria.

Clay bricks and blocks allow PCM placement in cavities or porous bodies, protecting the material while retaining replaceability. Experiments with PCM-incorporated clay bricks and location optimization in concrete blocks show that placement controls both charging and indoor heat release [79,80]. PCM close to the exterior may intercept solar gains but fail to discharge to the desired side; PCM close to the interior can regulate operative temperature but may be shielded by insulation. A layer centered within a massive wall can remain thermally inaccessible. Optimization therefore has to consider wall sequence, orientation, solar absorptance, insulation, night ventilation, and seasonal heat-flow reversal.

### 5.2. Timber and Lightweight Assemblies

Lightweight timber buildings have low inherent thermal mass and can benefit strongly from latent storage. PCM can be introduced into cross-laminated timber cavities, oriented strand board (OSB), furniture, or wood-derived porous scaffolds. Recent OSB development used high-density polyethylene to stabilize octadecane and additional additives to address bonding and flammability, producing lower heat flux than conventional OSB with comparable flammability characteristics [31]. Internal furniture can also provide distributed thermal mass without modifying the envelope [81]. Cross-laminated timber and wood-template composites extend this concept to engineered assemblies and durable porous supports [30,76].

The opportunity in timber must be balanced against combustibility, moisture, dimensional stability, and adhesive compatibility. PCM migration into wood or adhesive layers can change bond strength; fire-retardant treatments can alter porosity and storage loading. Because wood is anisotropic and hygroscopic, heat and moisture transport should be evaluated together. Component tests should include realistic joints, coatings, fasteners, and boundary conditions rather than ideal homogeneous specimens.

### 5.3. Roofs, Floors, Windows, and Solar-Coupled Envelopes

Roofs experience large solar excitation and can charge PCMs reliably in warm climates, but they also face high peak temperatures, UV exposure, and risk of remaining molten overnight. Nighttime regeneration through radiation or ventilation is therefore critical. Floors can couple to radiant heating or cooling and provide a large transfer area, although finish resistance can isolate the PCM. Window-integrated or water-flow-window systems can capture solar heat and transfer it to storage, offering controllable separation between optical gain and indoor release [82].

Solar walls and sunspaces provide predictable charging but must avoid unwanted summertime gains. Simulation of PCM storage walls in detached houses with sunspaces confirms that orientation, thickness, transition temperature, and climate interact strongly [83]. Solar-energy storage concrete panels and broader solar-thermal PCM systems show potential for space heating and cooling, yet source–storage–load temperature matching remains the governing condition [32,84,85]. In every envelope application, a complete daily cycle is preferable to permanent heat accumulation; otherwise the PCM saturates and loses its regulating function.

Recent component studies broaden this evidence from fixed wall layers to building-integrated floors, movable PCM layers, radiant floors, energy screw piles, exterior insulation systems, and macroencapsulated light-steel envelopes [75,86,87,88,89,90]. Novel wall configurations can moderate transient heat flow, but their benefit remains sensitive to placement and boundary conditions [91]. Together, these studies support component-specific optimization rather than transferring a nominal material result directly to a building assembly.

**Figure 3:**
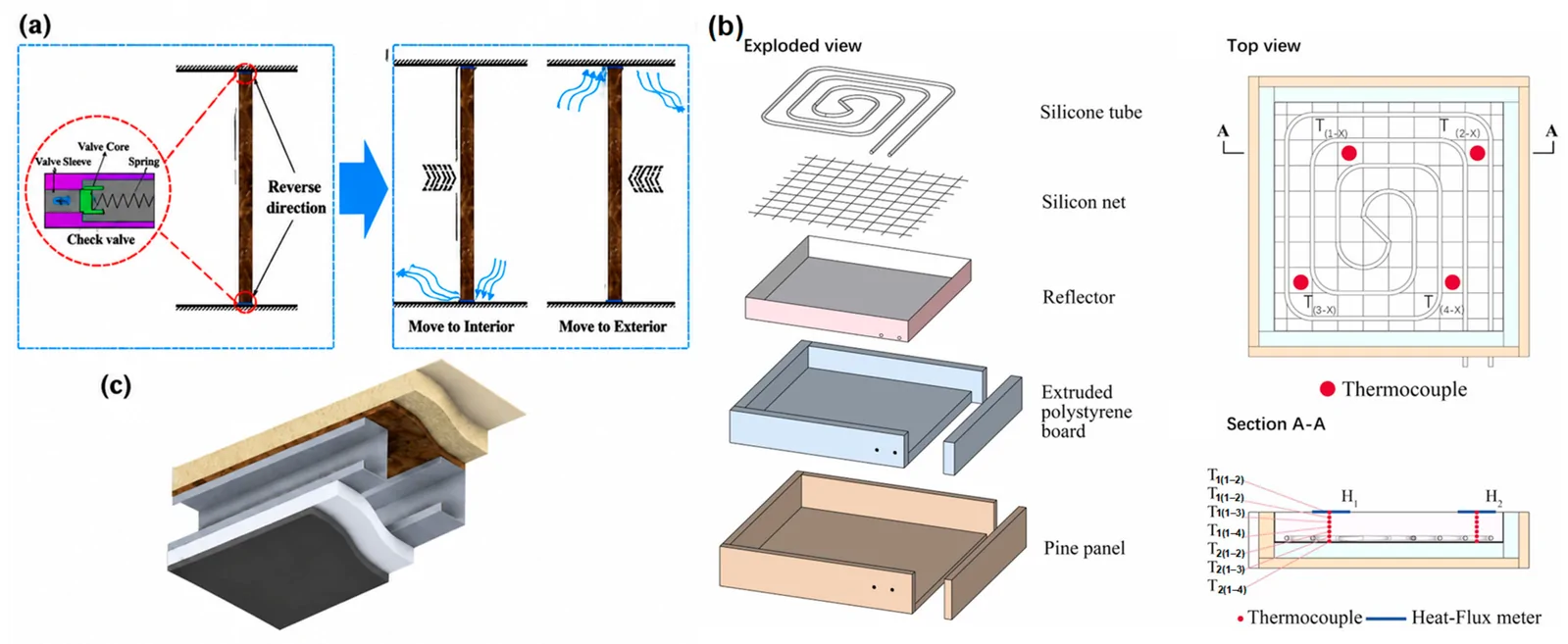
Examples of PCM integration into passive building components: (**a**) a self-adaptive movable PCM wall layer [87]; (**b**) an instrumented PCM radiant-floor module [88]; and (**c**) a layered light-steel-frame wall with macroencapsulated PCM. In panel (**c**), the outdoor-to-indoor assembly comprises finishing plaster and mineral-wool insulation, an OSB/steel-frame zone containing 15 mm aluminum PCM containers, and interior gypsum boards [75].

As shown in Figure 3, these configurations expose three distinct means of controlling thermal coupling: displacing the PCM position, distributing heat across a large radiant surface, and embedding replaceable latent-storage elements within a lightweight envelope. Their performance is governed not by PCM enthalpy alone but equally by contact resistance, layer sequence, sensor and control placement, and the availability of a daily regeneration pathway [75,87,88].

### 5.4. Design Synthesis for Passive Envelopes

Passive PCM design is a coupled sequence: define the comfort or surface-temperature target; obtain hourly temperature distributions for candidate locations; select a transition band crossed on the useful days; size latent capacity relative to the heat pulse; verify nighttime or off-peak regeneration; then optimize thickness, layer order, position, thermal resistance, and control schedule. Material choice, loading, placement, and set points must be selected together: a high-enthalpy formulation has little building value if service temperatures do not drive a useful transition or if the store cannot regenerate [13,92].

Table 2 summarizes representative eutectic, organic, bio-based, cementitious, and geopolymer PCM composites, including reported mass fractions, transition temperatures, enthalpies, conductivity, and cycling evidence. Table 4, by contrast, summarizes active storage and control studies; its system-level outcomes should not be interpreted as a material-formulation comparison.

A common interpretive error is to report a lower indoor peak temperature without accounting for a delayed or elevated nighttime temperature. PCMs redistribute heat rather than destroy it. Evaluation should therefore include peak magnitude, peak timing, overheating degree-hours, operative temperature, HVAC energy, and completeness of discharge. Here, a free-running test cell has no active HVAC control of indoor temperature during the assessment period, so its response is governed by weather, internal gains, and the envelope; a conditioned cell is maintained within specified HVAC set points, allowing energy use and peak-load effects to be quantified.

The comparison therefore retains temperature, heat flux, comfort, and energy as separate endpoints. It also includes negative and season-dependent evidence: in paired light-steel test buildings, macroencapsulated PCM reduced inner-surface peaks by 5–8 °C in favorable winter/spring periods, yet it was active during only 28% of wall and 20% of ceiling monitoring time and remained melted during summer, when overheating risk persisted. Favorable peak attenuation should not be generalized to annual energy savings [75].

## 6. Active Building Thermal Storage and Grid Interaction

### 6.1. HVAC-Integrated Storage

Active systems circulate air or liquid through a PCM store, making charge and discharge less dependent on incidental envelope temperatures. Applications include chilled-water and cool-energy storage, air-conditioning tanks, finned-tube heat exchangers, radiant systems, and ventilation preconditioning. A paraffin TES unit for cooling, partial tank charging studies, and a PCM thermal-management system illustrate the sensitivity of delivered capacity to flow rate, heat-exchanger geometry, initial state, and charge fraction. Evidence ranges from laboratory demonstrations to a 7000 L field installation that delivered 99 kWh per daily cycle (36% of nominal capacity) and a heat-pump/PCM control simulation reporting 42% lower operating cost and 31% lower energy use relative to its sensible-storage reference; these outcomes are not directly comparable because validation scales and system boundaries differ. Pumps, fans, valves, heat exchangers, and standby losses must be included in system efficiency [93,94,95,96,97].

The rate limitation of organic PCMs has motivated finned tubes and compact exchangers. Low-cost fin-tube designs and recent experimental PCM heat exchangers show that fin spacing, tube arrangement, flow direction, and natural convection dominate melt-front evolution [62,63]. Reported capacity should be accompanied by charging and discharging power, pressure drop, auxiliary energy, and state-of-charge uncertainty. A store with high total energy but insufficient discharge power cannot meet a building peak.

The expanded active-storage literature compares fin-stone hybrids, elliptic charging elements, shell-and-tube fins, plate exchangers, nanoparticles, composite plates, and macroencapsulated graphite stores [98,99,100,101,102,103,104,105,106]. Thermocline and cold-storage experiments additionally show that charge and discharge behavior must be characterized separately and under partial operating states [107,108,109]. These results reinforce power–capacity co-design and the need to report geometry, flow conditions, stratification, and auxiliary demand.

**Figure 4:**
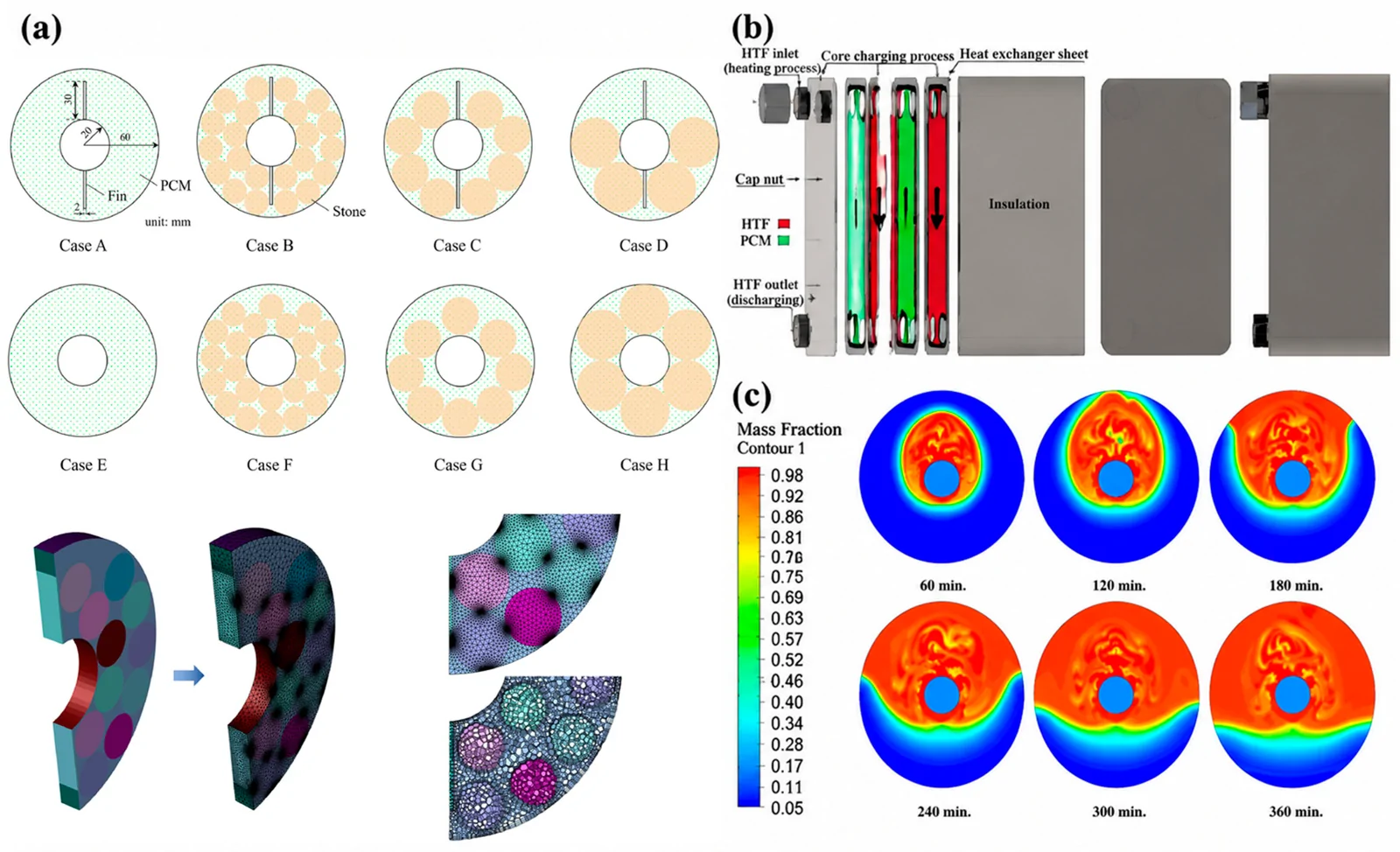
Representative active latent-heat storage and heat-transfer designs: (**a**) fin-stone hybrid arrangements and computational mesh [98]; (**b**) an alternating PCM/heat-transfer-fluid plate heat exchanger [103]; and (**c**) time-resolved liquid-fraction contours in a finned shell-and-tube store [104].

Figure 4a shows fin-stone hybrid arrangements and the computational mesh, panel (b) shows an alternating PCM/heat-transfer-fluid plate exchanger, and panel (c) shows time-resolved liquid-fraction contours in a finned shell-and-tube store. These designs illustrate complementary routes for increasing dischargeable power: conductive inclusions, shorter conduction paths, and fin-assisted melt-front development with natural convection. Because each enhancement displaces PCM volume or adds pumping, fabrication, and pressure-drop penalties, the appropriate objective is delivered power and usable capacity at the system level rather than minimum melting time alone [98,103,104].

### 6.2. Heat Pumps, Solar Systems, and Demand Response

Coupling PCM storage with heat pumps can shift operation to periods of higher coefficient of performance, lower electricity price, or lower grid carbon intensity. It can also reduce compressor cycling and allow smaller equipment when the storage reliably supplies peaks. Recent reviews show growing interest in heating systems and hybrid heat-pump configurations [7,110]. The storage transition temperature should be close enough to the load temperature to limit exergy loss but sufficiently separated to provide a practical heat-transfer driving force.

Demand-response value depends on control rather than capacity alone. A modeled building heating system with a heat pump and active PCM store used genetic-algorithm control to respond to time-varying electricity prices, reporting more than 40% cost savings and 30% consumption reduction relative to the study’s sensible-storage reference case [94]. These values are case-specific, but they illustrate the importance of co-optimizing building inertia, PCM state, weather, comfort constraints, equipment efficiency, and tariff. Model-predictive control can update this decision as forecasts change.

The quoted result is a case-specific numerical comparison, not a field-verified PCM-only effect. The model used a 110 kg PCM store (melting point 55 °C; latent heat 269.3 kJ kg^−1^), a 15 kW air-source heat pump, time-varying electricity prices, and genetic-algorithm scheduling while constraining indoor temperature to 21–23 °C during the day and 20–22 °C at night. Relative to the study’s sensible-storage reference, the reported reductions were 42% in operating cost and 31% in energy consumption; both storage medium and optimized control contributed to the outcome [94].

Solar-assisted PCM storage can bridge diurnal mismatch in domestic hot water, space heating, and absorption or evaporative cooling. Reviews of advanced solar PCM systems describe multiple collector and storage architectures [84,85]. The key constraints are charging temperature, stratification, stagnation safety, seasonal mismatch, and heat-exchanger effectiveness. High-temperature PCMs may store more useful heat but can lower collector efficiency and increase losses; low-temperature PCMs improve collection but may not meet the load without a heat pump.

Supplemental system studies extend this scope to solar trigeneration, salt-hydrate solar latent stores, residential micro-CHP, nanocomposite solar slurries, machine-tool heat recovery, and decentralized district-heating expansion [111,112,113,114,115,116]. Their common design requirement is temperature-level matching across source, store, and load; exergy and auxiliary-energy penalties should therefore be reported with the nominal storage capacity.

### 6.3. Economic and Operational Performance

A techno-economic study of cold storage in an office building demonstrates that economic outcome depends on tariff structure, plant size, operating strategy, and avoided peak demand rather than energy savings alone [93]. PCM storage may reduce electricity cost while slightly increasing total electricity because of additional heat-transfer losses or auxiliary power. Accordingly, energy, cost, emissions, and grid flexibility must be reported as separate outcomes. Carbon-aware control may charge at a different time from price-aware control, and both can conflict with equipment efficiency.

For the 7000 L full-scale salt-hydrate installation, 99 kWh was usable in a daily cycle compared with the supplier’s 275 kWh nominal capacity. Charging rates were 60–75% below design and more than 18 h of charging was impractical for daily service. The reported thermal cycling efficiency was 87%, falling to 76% when auxiliary electricity was included. Under the study tariff and optimization assumptions, 9804 SEK was the maximum investment compatible with a five-year payback; lifetime and maintenance cost were not reported [93].

Recent energy-economic and systematic optimization analyses examine hybrid sensible-latent fractions and broader sustainable supply configurations [117,118]. They confirm that the optimum PCM fraction is not a material constant: it changes with duty cycle, heat-exchanger effectiveness, tariff, system boundary, and the relative valuation of energy, exergy, cost, and emissions.

Operational robustness requires estimation of PCM state of charge. Temperature alone is ambiguous inside the phase-change interval and under hysteresis. Practical estimators can combine inlet and outlet temperatures, flow, heat-flux measurements, reduced-order enthalpy models, and periodic calibration. Fault detection should identify loss of capacity, leakage, sensor drift, blocked flow, and abnormal pressure drop. These functions are essential if PCM storage is to become a dependable grid-interactive building asset rather than an experimental add-on.

## 7. Characterization, Modeling, and Performance Assessment

### 7.1. Material-to-Component Characterization

DSC provides transition temperatures and enthalpy from milligram-scale samples, but results depend on sample mass, pan sealing, heating rate, prior thermal history, and integration method. For heterogeneous composites, a small sample may not represent PCM distribution. Large-scale calorimetry, T-history methods, guarded hot plates, heat-flow meters, and component heat-flux tests are needed to determine effective capacity and transfer rate. Reported conductivity must specify phase, temperature, density, orientation, and method because porous and graphitic composites can be strongly anisotropic.

A minimum characterization set should include melting and freezing onset, peak and end temperatures; enthalpy on both paths; supercooling and hysteresis; heat capacity outside transition; solid and liquid density; thermal conductivity across the relevant range; leakage and mass loss; chemical compatibility; and repeatability after cycling. For building products, this set expands to strength, stiffness, impact, fastener holding, dimensional change, water absorption, vapor transport, freeze–thaw, biological resistance, corrosion, VOC emissions, and fire response. Raw curves and uncertainty should be available, not only peak values.

The multiscale issue is increasingly explicit. Recent work combines thermal analysis, spectroscopy, microscopy, pore characterization, and component testing to understand how confinement and interfaces alter crystallization [73]. Such linkage is essential because an enthalpy loss can arise from PCM degradation, restricted crystallization, leakage, or sampling error; each requires a different remedy.

**Figure 5:**
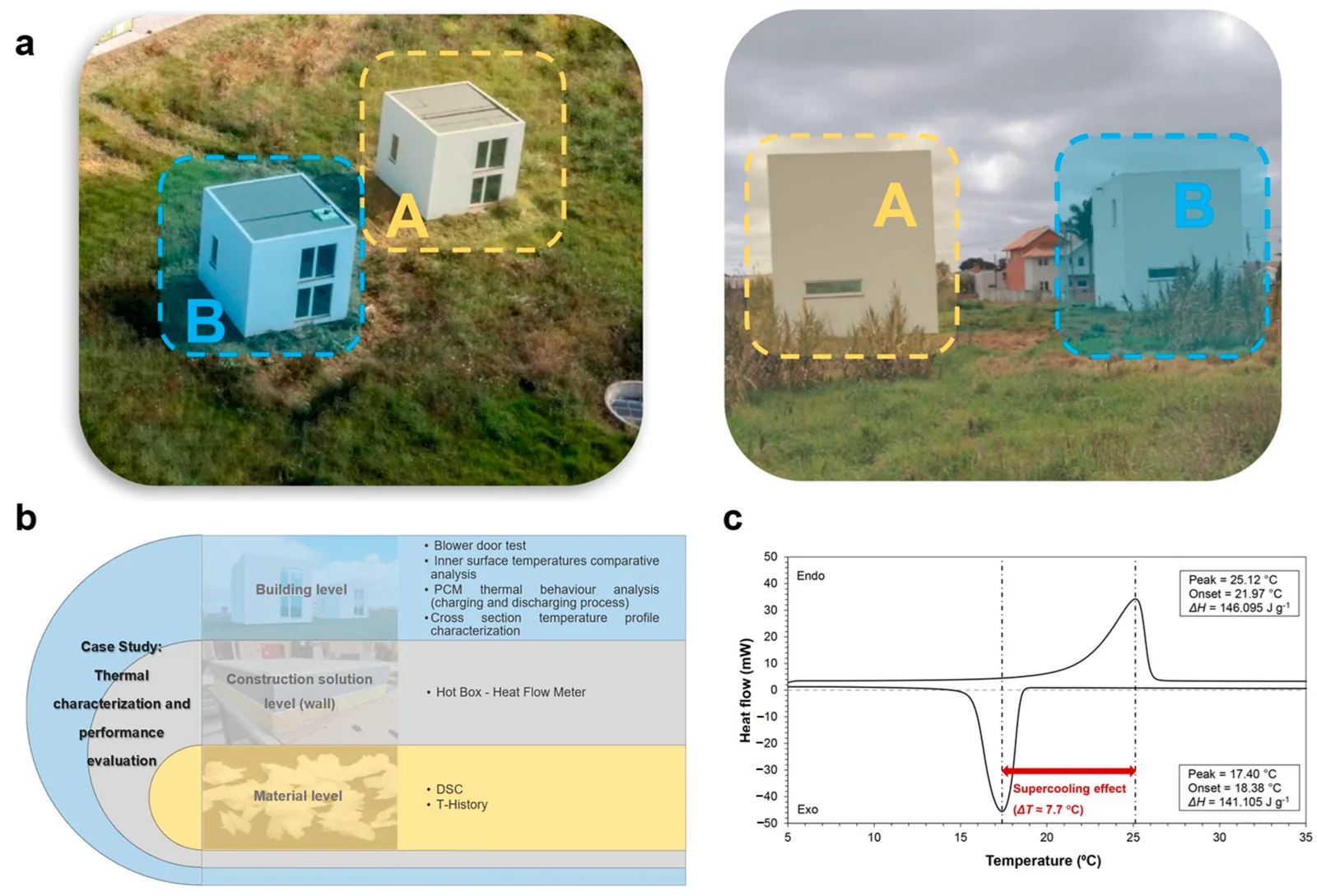
Multiscale validation of a macroencapsulated PCM building system: (**a**) paired reference and PCM-enhanced full-scale test buildings; (**b**) the linked material-, wall-, and building-level evaluation strategy; and (**c**) DSC evidence of melting/freezing enthalpy and supercooling [75].

Using one study across all panels makes the evidence chain explicit: laboratory calorimetry establishes the phase-transition window, wall-scale testing quantifies heat transfer through the assembly, and paired buildings determine whether the stored heat produces a measurable whole-building effect. This hierarchy reduces the risk of inferring field performance directly from milligram-scale enthalpy and supports traceable calibration of component and building models in Figure 5 [75].

The approximately 7.7 °C separation between the DSC melting and freezing peaks in Figure 5 is operationally important: regeneration requires the assembly to cool below the freezing range, not merely below the melting peak. The same full-scale study found that PCM remained melted during summer and was active only 28% of wall and 20% of ceiling monitoring time. Thus, laboratory hysteresis and the field regeneration shortfall form a consistent explanation for limited seasonal utilization [75].

### 7.2. Numerical Models

PCM simulation commonly uses apparent heat capacity, effective heat capacity, enthalpy, or enthalpy-porosity formulations. Whole-building programs often smear latent heat across a temperature interval; computational-fluid-dynamics models can resolve natural convection and moving melt fronts but are expensive. Hysteresis, supercooling, subcooling, temperature-dependent properties, anisotropic conductivity, and contact resistance are frequently simplified. Calibration against one thermal cycle does not ensure prediction under partial cycling or different rates.

Fast and accurate reduced-order approaches are important for annual building simulation and control [119]. A credible workflow calibrates material functions with independent data, validates a component model under several boundary conditions, then embeds a reduced model in whole-building analysis. Geometry, mesh and time-step independence, energy conservation, initial state, and convergence should be documented. Measured weather and occupancy should be used for field validation where available.

Apparent/effective-heat-capacity methods are efficient for whole-building simulation but smear latent heat over an assumed interval and usually require separate heating/cooling functions to represent hysteresis. Enthalpy or liquid-fraction methods conserve energy more transparently and handle partial states, whereas enthalpy-porosity CFD additionally resolves buoyancy and melt-front motion at much higher computational cost. Reduced-order maps are suitable for controls only within the temperature, flow, geometry, and state domain used to generate them. Across all approaches, heterogeneous composites, supercooling, contact resistance, anisotropy, and incomplete cycling require validation under more than one boundary condition.

### 7.3. Optimization and Decision Metrics

Optimization variables include PCM type, transition range, latent heat, loading, thickness, location, layer order, conductivity enhancer, heat-exchanger geometry, and control schedule. Objectives can conflict: annual energy, peak demand, discomfort, cost, emissions, embodied impact, and material mass. Multi-objective methods are therefore preferable to a single energy objective, particularly for nanoparticle-enhanced stores and building assemblies [15,120]. Results should report Pareto trade-offs and sensitivity to uncertain weather, occupancy, property degradation, and prices.

Table 5 compares modeling approaches used to represent PCM phase change from material/component calculations to annual building simulation. It summarizes phase-change formulations and inputs, validation evidence, computational demand, and limitations that govern model transfer and use in control.

Performance metrics must specify the system boundary, measurement period, initial and final states, heat-loss treatment, and whether the reported numerator is latent-only or total useful heat. Nominal latent capacity is defined as:

$$Q_{lat,nom}=m_{PCM}\times {\Delta H}_{pc}$$

For one identified charge–discharge event, latent utilization is defined as:

$$U_{lat}=\frac{Q_{lat,cycled}}{m_{PCM}\times {\Delta H}_{pc}}$$

Here, $m_{PCM}$ is the active PCM mass (kg), ΔH_pc_ is its reference latent enthalpy per unit mass (kJ kg^−1^), and $Q_{lat,cycled}$ is the latent heat actually released during the identified discharge event (kJ), estimated from a latent-only enthalpy or phase-fraction balance. $U_{lat}$ is dimensionless; under one complete cycle it should not exceed unity unless the reference basis or system boundary differs. Useful delivered capacity may include sensible and latent contributions and should not be labeled latent utilization. Thermal round-trip efficiency is defined as:

$$\eta_{th}=\frac{Q_{useful,dis}}{Q_{input,ch}}$$

Here, $Q_{useful,dis}$ is useful heat discharged and $Q_{input,ch}$ is heat supplied during charging, both evaluated at the same stated system boundary. Auxiliary electricity, pressure drop, controls, and pumping should be reported separately or included in an explicitly defined whole-system metric. Passive-envelope studies should attribute heat to the PCM layer through matched reference tests or calibrated counterfactual models rather than equating zone-load changes with PCM heat.

Uncertainty is seldom propagated across scales. PCM enthalpy, transition temperature, conductivity, weather, infiltration, occupant gains, and set points all vary. Reporting only a deterministic optimum encourages overfitting. Robust design should identify ranges in which a PCM remains beneficial and should test off-design years and future climate files. Characteristic-oriented ranking can then be combined with scenario-based building optimization to narrow the material search before expensive experiments [4].

## 8. Sustainability, Durability, and Commercial Translation

The environmental benefit of PCM systems depends on whether operational savings exceed the impacts associated with producing the PCM, shell, support, conductive additives, container, heat exchanger, and replacement materials. A service-based functional unit, such as one kilowatt-hour of useful thermal load shifted over the product lifetime, is more informative than one kilogram of composite. A high-enthalpy material that is rarely activated or fails prematurely may have a greater life-cycle impact than a lower-enthalpy material with high utilization and long-term stability. Bio-based and waste-derived formulations are promising, but their assessment must include processing energy, solvent use, additives, feedstock variability, transportation, maintenance, and end-of-life recovery [21,22,30,35,45,47,48,49].

Durability evidence remains a weak link. Accelerated cycling is usually performed under clean and fully reversible laboratory conditions, whereas buildings impose partial cycling, long dwell periods, humidity, contaminants, and mechanical restraint over decades. Service-life prediction needs identified degradation mechanisms and acceleration factors rather than a cycle count alone. Organic PCMs may oxidize or permeate shells; salt hydrates may dehydrate or segregate; conductive networks may settle or debond; capsules may fatigue; and porous supports may release PCM after cracking.

Useful storage can be improved by matching the transition interval to the duty cycle, increasing heat-transfer area or using conductive networks where conduction limits power, and selecting PCM fraction and geometry against delivered rather than nominal capacity. These measures must be balanced against inactive mass, cost, pressure drop, fire behavior, and reduced latent heat per unit composite. PCM behavior can be controlled through scheduled charging/discharging, cascaded or hybrid transition ranges, state-of-charge estimation, and forecast-based supervisory or model-predictive control; optimization should jointly consider comfort, useful energy, peak demand, cost, emissions, and the associated penalties [15,26,94,119,120].

Commercial products must satisfy construction workflows and codes. Required evidence includes batch consistency, storage and transport stability, installation tolerances, compatibility with adhesives and finishes, repairability, fire classification, emissions, and quality control. Macroencapsulated modules offer replaceability and clearer quality assurance, while dispersed microcapsules may be easier to install but are difficult to recover. Design for disassembly and transparent material passports could improve circularity and enable capacity verification at renovation.

Economic assessment should include material and fabrication cost, additional structure or containment, design labor, commissioning, controls, maintenance, replacement, salvage, and financing. Benefits may include reduced HVAC capacity, avoided demand charges, energy arbitrage, comfort, resilience, and renewable self-consumption. Because many benefits accrue to different stakeholders, business models and procurement specifications matter. Bankable deployment will require warranties expressed in retained usable capacity and power under specified conditions, analogous to electrochemical storage warranties.

The reviewed economic and environmental results cannot be pooled into a universal payback or break-even threshold. The Mardin-stone study reports USD 4.80 m^−2^ y^−1^ and 33.68 kg CO_2_ m^−2^ y^−1^ reductions under its specific construction, tariff, and emission assumptions; the full-scale cold-store study gives a 9804 SEK five-year-payback investment ceiling under a local tariff but lacks lifetime and maintenance data; and the 42% cost result for heat-pump storage is an operational simulation relative to a sensible-storage reference. Future comparisons should report installed cost, service life, maintenance, discount rate, tariff, emission factor, system boundary, and a service-based functional unit such as one kilowatt-hour of useful heat shifted over the product lifetime [33,93,94].

## 9. Critical Limitations of the Evidence Base and Future Perspectives

### 9.1. Methodological and Empirical Evidence Gaps

The findings synthesized in this review must be interpreted alongside key methodological and empirical boundaries. First, as a structured critical narrative review rather than a formal meta-analysis, the identification process relied on targeted keyword expansion and iterative citation chaining to capture cross-scale evidence. While this maximized topical coverage, it precluded a single, fixed initial-hit tally.

More critically, the underlying literature exhibits severe data heterogeneity. Published studies vary widely in sample mass, heating/cooling rates, enthalpy calculation baselines, phase-transition definitions, test durations, and operating setpoints. Fundamental engineering parameters—such as density changes across transitions, direction-dependent conductivity, degree of supercooling, long-term cycling retention, and parasitic auxiliary power—are frequently omitted (denoted as “N.R.” across Table 2, Table 3, Table 4 and Table 5). Crucially, the reporting of negative outcomes, failed regenerations, and performance degradation remains rare.

Finally, the current body of literature is heavily skewed toward milligram-scale laboratory characterizations and idealized numerical models. Multi-season matched field demonstrations, full-scale component aging under coupled hygrothermal stress, fire performance at the assembly scale, and empirical maintenance/installed-cost inventories remain scarce. Consequently, peak heat-flux or operating-cost reductions obtained under optimized or simulated conditions cannot be treated as universally transferable performance ceilings.

### 9.2. Application-First Future Perspectives

Despite substantial advances in PCM formulations and integration technologies, the translation of laboratory-scale thermal properties into reliable building performance remains a central challenge. High latent heat or favorable transition behavior measured under controlled conditions does not necessarily ensure effective operation within a building, where incomplete phase transitions, variable boundary conditions, contact resistance, moisture exposure, and seasonal changes can substantially reduce usable capacity. Future material development should therefore be guided by application-specific requirements, including the operating temperature range, available charging and discharging periods, required thermal power, dimensional constraints, fire and mechanical classifications, environmental exposure, target service life, and acceptable cost. Standardized databases that incorporate thermal hysteresis, supercooling, conductivity, degradation, compatibility, and uncertainty would support more reliable screening than selection based primarily on nominal enthalpy.

Composite engineering must resolve the persistent trade-offs among storage density, heat-transfer rate, containment, durability, safety, and manufacturability. Conductive networks, hierarchical porous supports, anisotropic biogenic scaffolds, fins, and metal foams can accelerate thermal response, but they also displace active PCM, increase embodied impacts, and introduce additional fabrication and recycling requirements. Material and heat-transfer structures should consequently be co-designed using system-level objectives such as activated capacity, delivered power, pressure drop, mechanical integrity, fire behavior, and life-cycle cost. Qualification procedures also need to extend beyond short-term thermal cycling by incorporating partial transitions, prolonged thermal dwell, humidity, ultraviolet exposure, freeze–thaw action, chemical attack, mechanical restraint, and assembly-scale fire testing. Such coupled aging protocols would provide a more defensible basis for predicting service life and identifying the dominant degradation mechanisms.

At the building and system scales, wider operating-temperature coverage and intelligent control provide important opportunities to improve annual PCM utilization. Cascaded, hybrid sensible–latent, and tunable storage configurations can accommodate variable climatic and operating conditions more effectively than a single fixed transition temperature, provided that additional complexity and control energy do not offset the thermal benefits. Active systems should integrate reduced-order enthalpy models, reliable state-of-charge estimation, weather and occupancy forecasts, and model-predictive control to coordinate comfort, energy cost, carbon intensity, renewable generation, and grid demand. Physics-informed surrogate models and machine-learning methods may accelerate design and operation, although their implementation should preserve energy conservation, quantify uncertainty, and remain robust under operating conditions beyond the training range. Shared benchmark cases and traceable validation datasets would further improve comparability among numerical approaches.

Commercial deployment will ultimately depend on continuous validation across material, component, building, and operational scales. Multi-season field demonstrations should use matched reference systems and report weather-normalized energy demand, peak-load reduction, thermal comfort, regeneration completeness, auxiliary consumption, maintenance requirements, degradation, and economic performance. Environmental assessment should adopt a service-based functional unit, such as useful thermal energy stored or shifted over the product lifetime, while accounting for processing, containment, conductive additives, replacement, transport, and end-of-life recovery. Modular and separable products, locally available renewable or waste-derived feedstocks, material passports, and recoverable containment systems can strengthen circularity without relying on nominally sustainable constituents alone. Performance warranties expressed in retained delivered capacity and discharge power under specified operating conditions would provide the technical and financial confidence required for PCMs to become dependable components of low-carbon and grid-interactive buildings.

## 10. Conclusions

This review shows that building value is governed by transition matching, activated fraction, heat-transfer rate, containment, and durability rather than nominal enthalpy alone. Across illustrative laboratory composites, reported latent heat spans 43.31–229.7 J g^−1^ and graphene addition has produced a 156.4% conductivity increase; however, inactive support, restricted crystallization, anisotropy, viscosity, displaced PCM mass, fire behavior, and mechanical penalties determine whether these gains survive product integration. These are formulation-specific findings, not universal performance ranges.

At component and building scales, the defensible evidence is more modest and strongly conditional. Concrete-based examples report peak-temperature reductions of approximately 1.0–1.81 °C, whereas a paired full-scale light-steel study reported 5–8 °C inner-surface attenuation in favorable winter/spring periods but only 20–28% monitored activation and no effective summer regeneration. A 7000 L field installation delivered 99 kWh per daily cycle, only 36% of nominal capacity, because charging power was substantially below design. These results demonstrate why geometry, location, climate, duration, baseline, and regeneration must accompany every performance claim.

The largest benefits remain predictions under specific controls: a modeled tunable wall produced up to 99% cooling-peak and 34% heating-peak heat-flow reductions, and a modeled heat-pump/PCM system produced 42% operating-cost and 31% energy reductions relative to its sensible-storage reference. These are design opportunities pending matched field validation. Priority research should standardize capacity and utilization definitions; test partial cycling, humidity, mechanics, and assembly-scale fire after aging; validate reduced models across off-design states; conduct multi-season reference-building trials; and report installed cost and life-cycle impact using explicit service-based boundaries. Together, these priorities move the field from property-led proof of concept toward service-verified building products.

## Figures and Tables

**Table 1:** Scope of representative prior reviews and the evidence gap addressed in the present work.

Review Scope	Principal Emphasis	Gap Addressed in the Present Review	Ref.
Broad recent building-PCM reviews	Materials, integration routes, and applications	Adds a common cross-scale evidence framework, explicit study-type labels, and an application-first decision pathway	[6,12]
Heating and hybrid applications	Heating-system and hybrid storage configurations	Connects active systems to passive envelopes, material constraints, control, durability, and validation	[7]
Passive building applications and envelopes	Envelope placement, indoor temperature, and energy response	Separates temperature, heat-flux, comfort, energy, and field-utilization outcomes and includes active storage	[8,13]
Cooling applications	Cold storage, cooling systems, and peak-load management	Adds heating, mixed-climate operation, containment, safety, and multi-season regeneration	[9,11]
Inorganic PCMs	Salt hydrates, supercooling, segregation, and stabilization	Compares inorganic, organic, eutectic, and bio-based options within the same building decision chain	[10]
Building PCM thermal energy storage across material, component, building, and system scales	Application-first synthesis of PCM chemistry, composite engineering, passive and active integration, modeling, control, durability, safety, economics, and life-cycle assessment	Links transition-temperature matching and containment to usable capacity, delivered power, regeneration, component and building validation, operational control, and service-based economic and environmental assessment; distinguishes laboratory, component, numerical, full-scale/field, and review evidence	This work

**Table 2:** Illustrative material and composite evidence normalized to common reporting variables; N.R. indicates not reported.

Formulation/Evidence Scale	Tpc (°C)	ΔH (J g^−1^)	k/Change	PCM Fraction	Cycling/Retained Evidence	Main Limitation	Ref.
PUA-coated salt hydrate/diatomite; laboratory composite	N.R.	102.6	N.R.	N.R.	Supercooling 0.5 °C; dehydration suppressed; protocol N.R.	Coating permeability, fire, and mechanical qualification N.R.	[18]
Gypsum/C18–C24; laboratory/component	26.74	43.31	N.R.	18 wt.%	1000 cycles; exact rate, dwell, and retained enthalpy N.R.	Large-panel aging and fire evidence N.R.	[27]
Capric–stearic acid/MMT/graphene; laboratory composite	N.R.	N.R.	+156.4% at 2 wt.% graphene	N.R.	Stable over 300 cycles; retained values N.R.	Dispersion, anisotropy, capacity penalty, and cost	[28]
Beeswax/graphene; laboratory composite	N.R.	+22.5% reported	2.89 W m^−1^ K^−1^ at 0.3 wt.%	≈99.7 wt.% PCM	N.R.	Mechanism, fire, oxidation, and feedstock variability	[25]
Methyl palmitate/palygorskite; laboratory composite	22.29–24.50	96.10 (freezing)	N.R.	46.32% absorption	300 cycles; full protocol/uncertainty N.R.	Moisture and component-scale validation N.R.	[29]
Paraffin/functionalized MWCNT; laboratory composite	28.46	229.7 (melting)	N.R.	N.R.	Less than 1% variation in phase-transition temperatures and enthalpies after 200 cycles	Dispersion, loading basis, cost, and fire evidence	[14]
BWT + PCM90/10	25.4 ± 0.6 °C	114.2 ± 1.8 J g^−1^	N.R.	N.R.; ≈30 wt.% PEG inactive/bound	No reported degradation after 1000 cycles; protocol N.R.	Inactive fraction, moisture, adhesive, and fire behavior	[30]
Octadecane/HDPE in OSB; product/component	26.4–28.4	N.R.	Lower heat flux than OSB	N.R.	N.R.	Assembly-scale moisture, adhesive, and fire aging	[31]
mPCM artificial aggregates in concrete panel; component study	26.3 melting/18.8 freezing (neat mPCM)	99.9 melting/111.8 freezing (neat mPCM)	N.R.	20 wt.% mPCM in AGGPCM20; selected for panel; panel loading N.R.	Core–shell aggregate design suppressed leakage; long-term cycling N.R.	AGGPCM20 aggregate crushing strength: 0.5 vs. 1.9 MPa for control (~74% lower); long-term weathering N.R.	[32]
Lauryl-alcohol/Mardin-stone shape-stabilized PCM in concrete; component study	19.95	46.6 composite; 46.1 after 750 cycles	−13.19% vs. reference concrete	20 wt.% PCM (reported formulation)	750 thermal cycles; no visible leakage at 55 °C for 3 h	Compressive strength decreased by 6.1 MPa; building validation is climate specific	[33]
Capric–palmitic eutectic in geopolymer foam (CA–PA@GF); laboratory composite	20.74	87.7 melting/86.3 freezing	N.R.	48.87% encapsulation ratio	≈100 cycles; enthalpy decrease ≈4%/3.5%; no visible leakage at 40 °C for 2 h	Mortar mechanics, assembly fire, and whole-building performance N.R.	[34]
Silica-stabilized capric–lauric PCM in low-carbon geopolymer mortar (GPm-20); composite	20.6 (fusion)	14.50 fusion; 14.16 crystallization	0.63 W m^−1^ K^−1^ at 0 °C; 0.60 at 50 °C	20 wt.% SiO_2_@PCM-1	Stable phase-change behavior after 50 heating–cooling cycles	Compressive strength 27.8 MPa; long-term moisture/field aging N.R.	[35]

**Table 3:** Illustrative building-component and construction-material evidence with climate/configuration, baseline, duration, and limitations. N.R. indicates not reported.

Application/Study Type	Climate or Configuration	Loading/Location	Baseline	Reported Outcome	Duration/Activation	Limit/Interpretation	Ref.
Tunable PCM in lightweight wall; numerical	Baltimore mixed climate; annual wall model; 23 °C cooling and 20 °C heating set points with ±1 °C load-shifting modulation	0.63 cm PCM behind drywall; default ΔH 50 kJ kg^−1^	Static and cascaded PCM walls	Up to 99% cooling-peak and 34% heating-peak heat-flow reduction; 27% annual utilization	One modeled year	Event-based wall heat-flow result, not annual building-energy savings; shoulder seasons not isolated	[26]
Gypsum/C18–C24; laboratory/component	Gypsum panel; climate N.R.	18 wt.%	Unmodified gypsum	Tpc 26.74 °C; ΔH 43.31 J g^−1^	1000 laboratory cycles	No occupied-building energy or comfort outcome	[27]
PCM-aggregate concrete panel; experiment	Solar-exposed panel	AGGPCM20 (melting peak 25.1 °C); panel location as reported	Reference concrete panel	Peak temperature reduced by ≈1 °C	Test duration N.R.	>50% compressive-strength loss constrains structural use	[32]
Mardin-stone PCM concrete; numerical/experimental framework	Room-scale configuration and stated environmental/economic boundary	Loading N.R.	Reference construction	Room-center temperature reduced up to 1.81 °C; 33.68 kg CO_2_ m^−2^ y^−1^ and USD 4.80 m^−2^ y^−1^ reductions	Annual assessment	Results depend on local baseline, tariff/emission factor, and system boundary	[33]
PCM-integrated CLT wall; numerical	Building-envelope model; climate/configuration as reported	PCM in CLT assembly	Conventional CLT	Annual energy use reduced up to 11.76%	Annual simulation	Prediction; field validation and seasonal regeneration N.R.	[76]
Macroencapsulated salt hydrate in light-steel wall/ceiling; paired full-scale field study	Mediterranean climate; matched reference and enhanced buildings	Macroencapsules in wall and ceiling	Paired reference building	Inner-surface peaks reduced 5–8 °C in favorable winter/spring periods	Multi-season monitoring; active only 28% (wall) and 20% (ceiling)	PCM remained melted in summer and overheating risk persisted	[75]
Organic PCM in geopolymer foam; laboratory material/component	Low-temperature building TES	Vacuum-impregnated capric–palmitic eutectic; 48.87% encapsulation ratio	Neat constituents/reference foam	No wall/panel or occupied-building performance reported; material properties are summarized in Table 2.	Laboratory cycling only (≈100 cycles; see Table 2)	Construction-material proof of concept; mortar mechanics, assembly fire, and field validation N.R.	[34]
Bio-based PCM in low-carbon geopolymer mortar; laboratory material/component	Mortar integration	GPm-20 with 20 wt.% silica-stabilized capric–lauric PCM	Reference geopolymer mortar	No room/panel or building energy/comfort outcome reported; material properties are summarized in Table 2.	50 laboratory heating-cooling cycles (see Table 2)	Long-term moisture, assembly fire, and building validation N.R.	[35]

**Table 4:** Illustrative active storage and control evidence; outcomes are not directly comparable without a common operating boundary. N.R. indicates not reported.

System/Study Type	Operating Range/Inventory	Usable Capacity/Power	Baseline/Control	Outcome and Auxiliaries	Duration	Principal Limitation	Ref.
Office-building cold store; full-scale field + calibrated model	7000 L commercial salt hydrate; 9380 kg; charge inlet ≈ 8 °C; discharge loop ≈ 14–16 °C	99 kWh daily usable vs. 275 kWh nominal (36%); charging rate 60–75% below design	District cooling; MILP scheduling	87% thermal-cycle efficiency; 76% including auxiliary electricity; 5-year-payback investment limit 9804 SEK under stated tariff	Campaign plus annual optimization	>18 h charging infeasible for daily service; lifetime and maintenance cost N.R.	[93]
Heat pump + active PCM store; numerical control study	110 kg PCM; Tm 55 °C; ΔH 269.3 kJ kg^−1^; 15 kW heat pump	Capacity/power reported through model; standalone measured field values N.R.	GA control vs. sensible-TES reference; time-varying electricity price	42% operating-cost and 31% energy reduction; comfort constrained to 21–23 °C daytime and 20–22 °C nighttime	Case-study horizon	Benefits combine PCM and optimized control; no field demonstration	[94]
Paraffin store for building cooling; experiment	Operating temperatures as reported	Measured charge/discharge behavior	No-storage/reference condition as reported	Cooling-oriented technical performance	Laboratory campaign	Installed cost, auxiliary electricity, and multi-season performance N.R.	[95]
Cool-energy PCM thermal management; experiment	PCM/system range as reported	Measured thermal response	Reference configuration as reported	Building-cooling feasibility	Laboratory campaign	Long-term degradation and matched building validation N.R.	[96]
Partially charged PCM tank in building cooling; numerical/experimental analysis	Partial-state operation	Capacity depends on initial state and discharge fraction	Full/partial charging scenarios	Shows the importance of state history for delivered performance	Study-specific cycles	Cross-study comparison requires common states, losses, and auxiliary accounting	[97]

**Table 5:** Critical comparison of PCM modeling approaches for material-to-building analysis.

Model Approach	Typical Scale/Use	Phase-Change Representation and Inputs	Validation/Reported Accuracy	Computational Demand and Limitation	Ref.
Apparent/effective heat capacity	Envelope-scale annual wall heat-flow simulation	Latent heat distributed over a temperature interval; c_app(T), k(T), density, boundary conditions	Wall model reported RMSE < 1 °C against prior experimental data	Low-to-moderate cost; sensitive to interval choice and often omits hysteresis, supercooling, and partial-cycle history	[26]
Enthalpy/liquid-fraction finite volume	Tank/component simulation	H(T) or liquid fraction with sensible and latent terms; mesh, time step, heat-transfer coefficients	Calibrated to the full-scale store; a 9600-node, 5 s model was mesh/time-step independent; aggregate error N.R.	Moderate cost; <3 min per run on the reported laptop; uncertain internal sensors and material properties limit inference	[93]
Enthalpy-porosity CFD	Detailed melt-front, natural-convection, and heat-exchanger design	Energy and momentum equations with mushy-zone treatment, buoyancy, geometry, and temperature-dependent properties	Case-specific experimental or grid-independence checks; common error metric N.R.	High cost; parameters for mushy zone, contact resistance, and three-dimensional geometry can dominate results	[98,101,102]
Resistance-capacitance/performance-map reduced model	System controls and multi-day/annual building simulation	PCM heat-exchanger maps generated from a generalized detailed model and coupled to Modelica/EnergyPlus loads	Average absolute percentage error 2–4% against detailed PCM-HX simulations	At least 1800× faster than the generalized RC simulations used as source data; validity is bounded by the map domain	[119]
Optimization/surrogate workflow	Material screening and multi-objective design	Characteristic-oriented ranking or surrogate response linked to energy, comfort, cost, and emissions	Independent test-set and off-design validation inconsistently reported	Efficient for search but can violate conservation or extrapolate poorly without physics and uncertainty constraints	[4,120]

## Data Availability

No new data were created or analyzed in this study. Data sharing is not applicable to this article.

## References

[1] Khudhair A.M., Farid M.M. (2004). A review on energy conservation in building applications with thermal storage by latent heat using phase change materials. Energy Convers. Manag..

[2] de Gracia A., Cabeza L.F. (2015). Phase change materials and thermal energy storage for buildings. Energy Build..

[3] Zhou D., Zhao C.Y., Tian Y. (2012). Review on thermal energy storage with phase change materials (PCMs) in building applications. Appl. Energy.

[4] Awan M.B., Ma Z., Lin W., Pandey A.K., Tyagi V.V. (2023). A characteristic-oriented strategy for ranking and near-optimal selection of phase change materials for thermal energy storage in building applications. J. Energy Storage.

[5] Barreneche C., Navarro H., Serrano S., Cabeza L.F., Fernández A.I. (2014). New Database on Phase Change Materials for Thermal Energy Storage in Buildings to Help PCM Selection. Energy Procedia.

[6] Sharshir S.W., Joseph A., Elsharkawy M., Hamada M.A., Kandeal A.W., Elkadeem M.R., Kumar Thakur A., Ma Y., Eid Moustapha M., Rashad M. (2023). Thermal energy storage using phase change materials in building applications: A review of the recent development. Energy Build..

[7] Faraj K., Khaled M., Faraj J., Hachem F., Castelain C. (2021). A review on phase change materials for thermal energy storage in buildings: Heating and hybrid applications. J. Energy Storage.

[8] Ben Romdhane S., Amamou A., Ben Khalifa R., Saïd N.M., Younsi Z., Jemni A. (2020). A review on thermal energy storage using phase change materials in passive building applications. J. Build. Eng..

[9] Masood U., Haggag M., Hassan A., Laghari M. (2023). A Review of Phase Change Materials as a Heat Storage Medium for Cooling Applications in the Built Environment. Buildings.

[10] Junaid M.F., Rehman Z.u., Čekon M., Čurpek J., Farooq R., Cui H., Khan I. (2021). Inorganic phase change materials in thermal energy storage: A review on perspectives and technological advances in building applications. Energy Build..

[11] Faraj K., Khaled M., Faraj J., Hachem F., Castelain C. (2020). Phase change material thermal energy storage systems for cooling applications in buildings: A review. Renew. Sustain. Energy Rev..

[12] Hassan F., Jamil F., Hussain A., Ali H.M., Janjua M.M., Khushnood S., Farhan M., Altaf K., Said Z., Li C. (2022). Recent advancements in latent heat phase change materials and their applications for thermal energy storage and buildings: A state of the art review. Sustain. Energy Technol. Assess..

[13] Rathore P.K.S., Gupta N.K., Yadav D., Shukla S.K., Kaul S. (2022). Thermal performance of the building envelope integrated with phase change material for thermal energy storage: An updated review. Sustain. Cities Soc..

[14] Hayat M.A., Chen Y., Yang Y., Li L., Bevilacqua M. (2024). Enhancing thermal energy storage in buildings with novel functionalised MWCNTs-enhanced phase change materials: Towards efficient and stable solutions. Therm. Sci. Eng. Prog..

[15] Aydın K. (2026). Multi-objective optimization of melting time and thermal energy storage performance of nanoparticle-enhanced phase change materials embedded in metal foam. J. Energy Storage.

[16] Sarı A., Karaipekli A. (2012). Fatty acid esters-based composite phase change materials for thermal energy storage in buildings. Appl. Therm. Eng..

[17] Sarcinella A., Frigione M. (2024). Selection of PEG-Matrix Combinations to Achieve High Performance Form-Stable Phase Change Materials for Building Applications. Coatings.

[18] Xie N., Niu J., Zhong Y., Gao X., Zhang Z., Fang Y. (2020). Development of polyurethane acrylate coated salt hydrate/diatomite form-stable phase change material with enhanced thermal stability for building energy storage. Constr. Build. Mater..

[19] Yang Y., Ma Y., Lian P., Zhang L., Chen Y., Sheng X. (2025). Advanced engineering of binary eutectic hydrate composite phase change materials with enhanced thermophysical performance for high-efficiency building thermal energy storage. Sol. Energy Mater. Sol. Cells.

[20] Sang G., Zeng H., Guo Z., Cui H., Zhang Y., Cui X., Zhang L., Han W. (2022). Studies of eutectic hydrated salt/polymer hydrogel composite as form-stable phase change material for building thermal energy storage. J. Build. Eng..

[21] Hammami N., Liu L., Trovalet L., Bigot D., Malet-Damour B., Habas J.P. (2023). Can vegetable oils be used as sustainable phase change materials for thermal energy storage in building?. Mater. Today Sustain..

[22] Malode S.J., Shetti N.P. (2025). Thermal energy storage systems using bio-based phase change materials: A comprehensive review for building energy efficiency. J. Energy Storage.

[23] Boussaba L., Makhlouf S., Foufa A., Lefebvre G., Royon L. (2019). vegetable fat: A low-cost bio-based phase change material for thermal energy storage in buildings. J. Build. Eng..

[24] Wang X., Zhang G., Sun Y., Liu C., Rao Z. (2026). Design and characterization of advanced wood-based beeswax composite phase change materials for passive thermal regulation in energy-efficient buildings. Constr. Build. Mater..

[25] Amin M., Putra N., Kosasih E.A., Prawiro E., Luanto R.A., Mahlia T.M.I. (2017). Thermal properties of beeswax/graphene phase change material as energy storage for building applications. Appl. Therm. Eng..

[26] Kishore R.A., Booten C., Bianchi M.V.A., Vidal J., Jackson R. (2022). Evaluating cascaded and tunable phase change materials for enhanced thermal energy storage utilization and effectiveness in building envelopes. Energy Build..

[27] Karaipekli A., Sarı A., Biçer A. (2016). Thermal regulating performance of gypsum/(C18–C24) composite phase change material (CPCM) for building energy storage applications. Appl. Therm. Eng..

[28] Jin W., Jiang L., Chen L., Gu Y., Guo M., Han L., Ben X., Yuan H., Lin Z. (2021). Preparation and characterization of capric-stearic acid/montmorillonite/graphene composite phase change material for thermal energy storage in buildings. Constr. Build. Mater..

[29] Zhang H., Zhang L., Li Q., Huang C., Guo H., Xiong L., Chen X. (2019). Preparation and characterization of methyl palmitate/palygorskite composite phase change material for thermal energy storage in buildings. Constr. Build. Mater..

[30] Foster K.E.O., Magalindan B., Perruci G.F., Li Y., Zheng Q., Lynch K., Srubar W.V., Chen X., Booten C., Lu H. (2025). Wood template-supported phase change material composites for durable and form-stable thermal energy storage in buildings. Mater. Today Energy.

[31] Hasan L.M., Khanna S. (2026). Development and Evaluation of an Integrated Phase Change Material Oriented Strand Board for Thermal Energy Storage in Building Walls. Energies.

[32] Drissi S., Ling T.-C., Mo K.H. (2020). Thermal performance of a solar energy storage concrete panel incorporating phase change material aggregates developed for thermal regulation in buildings. Renew. Energy.

[33] Ustaoğlu A., Torlaklı H., Cay V.V., Kocyiğit Ş., Erdoğmuş E., Hekimoğlu G., Sarı A., Gencel O. (2025). Development of Mardin Stone-Based Shape-Stabilized phase change material concretes for sustainable thermal energy storage in buildings. Energy Build..

[34] Moulakhnif K., Moujoud Z., El Majd A., Ousaleh H.A., Faik A., Sair S., El Bouari A. (2025). Eutectic organic phase change materials integrated into inorganic geopolymer foam for low-temperature thermal energy storage in building applications. Process Saf. Environ. Prot..

[35] Dihaji H., Bouglada S., Kacem M., Essaddek A., Bih L., Sair S., Haily E.L.M. (2026). Silica shape-stabilized bio-based phase change materials integrated into low-carbon geopolymer mortars for thermal energy storage in buildings. Constr. Build. Mater..

[36] Shumuye E.D., Fame C.M., Mehrpay S., Riaz M.H., Sadowski Ł., Ueda T., Dong B., Fang G. (2026). A review on phase change material encapsulation and integration in building structures for thermal energy storage. Results Eng..

[37] Das R., Siva Ranjani Gandhi I., Muthukumar P. (2022). Use of agglomerated Micro-encapsulated phase change material in cement mortar as thermal energy storage material for buildings. Mater. Today Proc..

[38] Su W., Darkwa J., Zhou T., Du D., Kokogiannakis G., Li Y., Wang L., Gao L. (2023). Development of Composite Microencapsulated Phase Change Materials for Multi-Temperature Thermal Energy Storage. Crystals.

[39] Ennamri A., Bencaid J., Draoui K., Ouarga A., Oualid H.A. (2025). Development of a new kaolinite/phase change material (PCM) composite for latent heat thermal energy storage in building applications. Mater. Sci. Eng. B.

[40] Ranjbar S.G., Roudini G., Barahuie F. (2020). Fabrication and characterization of phase change material-SiO_2_ nanocomposite for thermal energy storage in buildings. J. Energy Storage.

[41] Baskar I., Chellapandian M., Jeyasubramanian K. (2022). LA-PA eutectic/nano-SiO_2_ composite phase change material for thermal energy storage application in buildings. Constr. Build. Mater..

[42] Ali Memon S., Yiu Lo T., Shi X., Barbhuiya S., Cui H. (2013). Preparation, characterization and thermal properties of Lauryl alcohol/Kaolin as novel form-stable composite phase change material for thermal energy storage in buildings. Appl. Therm. Eng..

[43] Tang F., Cao L., Fang G. (2014). Preparation and thermal properties of stearic acid/titanium dioxide composites as shape-stabilized phase change materials for building thermal energy storage. Energy Build..

[44] Yue X., Zhang R., Jin X., Zhang X., Bao G., Qin D. (2023). Bamboo-derived phase change material with hierarchical structure for thermal energy storage of building. J. Energy Storage.

[45] Mohaisen K.O., Hasan Zahir M., Maslehuddin M., Al-Dulaijan S.U. (2022). Development of a shape-stabilized phase change material utilizing natural and industrial byproducts for thermal energy storage in buildings. J. Energy Storage.

[46] Wen R., Liu Y., Yang C., Zhu X., Huang Z., Zhang X., Gao W. (2021). Enhanced thermal properties of stearic acid/carbonized maize straw composite phase change material for thermal energy storage in buildings. J. Energy Storage.

[47] Jin D., Yong Choi J., Nam J., Yuk H., Kim S. (2024). Innovative building materials by upcycling clothing waste into thermal energy storage matrix with phase change materials. Waste Manag..

[48] Cao X., Zhang R., Zhang N., Chen L., Chen D., Li X. (2023). Performance improvement of lauric acid-1-hexadecanol eutectic phase change material with bio-sourced seashell powder addition for thermal energy storage in buildings. Constr. Build. Mater..

[49] El Majd A., Sair S., Ousaleh H.A., Moulakhnif K., Krida S., Younsi Z., Belouaggadia N., El Bouari A. (2025). Phosphogypsum valorization for sustainable building applications: Leveraging shape-stabilized phase change materials towards advanced thermal energy storage in paints. Constr. Build. Mater..

[50] Das M., Stachewicz U. (2024). 3D carbon based phase change composites: A review on progress in fabrication strategies, thermal energy storage-conversion efficacy, prototypes, numerical models and applications. J. Energy Storage.

[51] Palacios A., Navarro-Rivero M.E., Zou B., Jiang Z., Harrison M.T., Ding Y. (2023). A perspective on Phase Change Material encapsulation: Guidance for encapsulation design methodology from low to high-temperature thermal energy storage applications. J. Energy Storage.

[52] Buonomo B., Rita Golia M., Manca O., Nardini S. (2024). A review on thermal energy storage with phase change materials enhanced by metal foams. Therm. Sci. Eng. Prog..

[53] Guo Z., Qiao J., Liu X., Lin F., Liu M., Fang M., Huang Z., Zhang X., Min X. (2024). Advances in mineral-based composite phase change materials for energy storage: A review. Green Smart Min. Eng..

[54] Zhao Y., Zhang K., Min X., Xiao J., Xu Z., Huang Z., Liu Y.g., Wu X., Fang M. (2022). Graphene aerogel stabilized phase change material for thermal energy storage. Case Stud. Therm. Eng..

[55] Ren F., Hou J., Shi C., Li Z., Sun B., Zhang K., Feng Y., Tian W. (2024). Hydrophilicity regulation of carbon nanotubes as phase-change materials for thermal energy storage. J. Mater. Res. Technol..

[56] Zhu Q., Ong P.J., Goh S.H.A., Yeo R.J., Wang S., Liu Z., Loh X.J. (2024). Recent advances in graphene-based phase change composites for thermal energy storage and management. Nano Mater. Sci..

[57] Simonsen G., Ravotti R., O’Neill P., Stamatiou A. (2023). Biobased phase change materials in energy storage and thermal management technologies. Renew. Sustain. Energy Rev..

[58] Mohtasim M.S., Das B.K. (2024). Biomimetic and bio-derived composite Phase Change Materials for Thermal Energy Storage applications: A thorough analysis and future research directions. J. Energy Storage.

[59] Yazdani McCord M.R., Kankkunen A., Chatzikosmidou D., Seppälä A., Seppälä J., Baniasadi H. (2024). Polypyrrole-modified flax fiber sponge impregnated with fatty acids as bio-based form-stable phase change materials for enhanced thermal energy storage and conversion. J. Energy Storage.

[60] Cui S., Kishore R.A., Kolari P., Zheng Q., Kaur S., Vidal J., Jackson R. (2023). Model-driven development of durable and scalable thermal energy storage materials for buildings. Energy.

[61] Akamo D.O., Kumar N., Li Y., Pekol C., Li K., Goswami M., Hirschey J., LaClair T.J., Keffer D.J., Rios O. (2023). Stabilization of low-cost phase change materials for thermal energy storage applications. iScience.

[62] Tamraparni A., Rendall J., Shen Z., Hun D., Shrestha S. (2025). Experimental investigation on phase change material–based finned tube heat exchanger for thermal energy storage and building envelope thermal management. Appl. Therm. Eng..

[63] Rendall J., Tamraparni A., Shen Z., Hun D., Shrestha S. (2024). Low-cost fin-tube heat exchanger design for building thermal energy storage using phase change material. Int. Commun. Heat Mass Transf..

[64] Mert H.H., Okkay H., Mert M.S. (2022). Form-stable n-hexadecane/zinc borate composite phase change material for thermal energy storage applications in buildings. Sustain. Energy Technol. Assess..

[65] Chen F., Wolcott M. (2015). Polyethylene/paraffin binary composites for phase change material energy storage in building: A morphology, thermal properties, and paraffin leakage study. Sol. Energy Mater. Sol. Cells.

[66] Santhanam K., Ramadoss R. (2025). Integration of organic phase change materials into alccofine-air lime mortar for building thermal energy storage applications. J. Energy Storage.

[67] Giro-Paloma J., Barreneche C., Maldonado-Alameda A., Royo M., Formosa J., Fernandez A.I., Chimenos J.M. (2019). Alkali-Activated Cements for TES Materials in Buildings’ Envelops Formulated With Glass Cullet Recycling Waste and Microencapsulated Phase Change Materials. Materials.

[68] Sarcinella A., Aguiar J.L.B., Lettieri M., Cunha S., Frigione M. (2020). Thermal Performance of Mortars Based on Different Binders and Containing a Novel Sustainable Phase Change Material (PCM). Materials.

[69] Li D., Tang Y., Zuo X., Zhao X., Zhang X., Yang H. (2023). Preparation of hierarchical porous microspheres composite phase change material for thermal energy storage concrete in buildings. Appl. Clay Sci..

[70] Zanjani M.M.K., Ferreira S.R., Lagazzo A., Basbus J.F., Fachinotti V.D., Caggiano A. (2026). Thermal energy storage-enhanced self-leveling mortars made of pre-placed perlite aggregates filled with bio-based phase change materials. Energy Build..

[71] Erkizia E., Strunz C., Dauvergne J.L., Goracci G., Peralta I., Serrano A., Ortega A., Alonso B., Zanoni F., Düngfelder M. (2024). Study of paraffinic and biobased microencapsulated PCMs with reduced graphene oxide as thermal energy storage elements in cement-based materials for building applications. J. Energy Storage.

[72] Sarcinella A., Barroso de Aguiar J.L., Jesus C., Frigione M. (2023). Thermal properties of PEG-based form-stable Phase Change Materials (PCMs) incorporated in mortars for energy efficiency of buildings. J. Energy Storage.

[73] Tamraparni A., Shen Z., Rendall J., Hun D., Shrestha S. (2026). Multiscale characterization of phase change materials for building thermal energy storage applications. J. Build. Eng..

[74] Sarcinella A., Frigione M. (2026). Phase Change Materials in Lime-Based Mortars for the Energy Efficiency of Historic Buildings: State-of-the-Art and Prospects. Coatings.

[75] Gonçalves M., Figueiredo A., Almeida R.M.S.F., Vicente R., Samagaio A., Kośny J. (2024). Thermal performance evaluation of a light steel framing building with macroencapsulated phase change materials in a Mediterranean climate. Energy Build..

[76] Park J., Chang S.J., Kim S. (2026). Enhancing thermal performance of cross-laminated timber using phase change materials for energy efficient building envelopes. Appl. Therm. Eng..

[77] Lachheb M., Karkri M., Nasrallah S.B. (2015). Development and thermal characterization of an innovative gypsum-based composite incorporating phase change material as building energy storage system. Energy Build..

[78] Kumar N., Rathore P.K.S., Sharma R.K., Gupta N.K. (2023). Integration of lauric acid/zeolite/graphite as shape stabilized composite phase change material in gypsum for enhanced thermal energy storage in buildings. Appl. Therm. Eng..

[79] Gupta M.K., Rathore P.K.S., Kumar R., Gupta N.K. (2023). Experimental analysis of clay bricks incorporated with phase change material for enhanced thermal energy storage in buildings. J. Energy Storage.

[80] Patel B., Rathore P.K.S., Gupta N.K., Sikarwar B.S., Sharma R.K., Kumar R., Pandey A.K. (2023). Location optimization of phase change material for thermal energy storage in concrete block for development of energy efficient buildings. Renew. Energy.

[81] Johra H., Heiselberg P. (2017). Influence of internal thermal mass on the indoor thermal dynamics and integration of phase change materials in furniture for building energy storage: A review. Renew. Sustain. Energy Rev..

[82] Yamaç H.İ., Koca A. (2021). Experimental investigation of water flow window system and numerical modeling of solar thermal energy storage with phase change materials on the way of nearly zero energy buildings. J. Energy Storage.

[83] Vukadinović A., Radosavljević J., Đorđević A. (2020). Energy performance impact of using phase-change materials in thermal storage walls of detached residential buildings with a sunspace. Sol. Energy.

[84] Tyagi V.V., Chopra K., Kalidasan B., Chauhan A., Stritih U., Anand S., Pandey A.K., Sarı A., Kothari R. (2021). Phase change material based advance solar thermal energy storage systems for building heating and cooling applications: A prospective research approach. Sustain. Energy Technol. Assess..

[85] Zhou Z., Zhang Z., Zuo J., Huang K., Zhang L. (2015). Phase change materials for solar thermal energy storage in residential buildings in cold climate. Renew. Sustain. Energy Rev..

[86] Wang T., Wang H., Wang A., Lahdelma R., Wang G., Han J. (2024). A comprehensive review on building integrated phase change floors with phase change materials for energy storage and indoor environment control. J. Energy Storage.

[87] Li W., Rahim M., Wu D., El Ganaoui M., Bennacer R. (2024). Dynamic integration of phase change material in walls for enhancing building thermal performance—A novel self-adaptive method for moving PCM layer. Energy Convers. Manag..

[88] Li G., Zhang P., Xu G., Gantumur T. (2024). Experimental study of advanced phase change materials to optimise the thermal storage performance of radiant floors: Towards a low-carbon energy system for grassland pastoral settlements. Constr. Build. Mater..

[89] Bandeira Neto L.A., Fei W., Narsilio G.A. (2024). Improving thermal performance of groups of energy screw piles with phase change materials. Appl. Therm. Eng..

[90] Wi S., Kim Y.U., Chang S.J., Berardi U., Kim S. (2024). Novel exterior insulation finishing: Enhancing building energy efficiency and flame-retardancy through thermal storage and fire propagation prevention. Case Stud. Therm. Eng..

[91] He Y., Li S., Ariffin M.M., Abu-Hamdeh N.H., Karimipour A., Hatamleh R.I., Viet P.H.H., Karimipour A. (2024). New structure for better thermal resistance of building with phase change material: Response to milder thermal change than common walls. Case Stud. Therm. Eng..

[92] Fachinotti V.D., Bre F., Mankel C., Koenders E.A.B., Caggiano A. (2020). Optimization of Multilayered Walls for Building Envelopes Including PCM-Based Composites. Materials.

[93] Tan P., Lindberg P., Eichler K., Löveryd P., Johansson P., Kalagasidis A.S. (2020). Thermal energy storage using phase change materials: Techno-economic evaluation of a cold storage installation in an office building. Appl. Energy.

[94] Nunna A.C., Zong Y., Georges L., You S. (2023). Demand response with active phase change material based thermal energy storage in buildings. Energy Rep..

[95] Dogkas G., Koukou M.K., Konstantaras J., Pagkalos C., Lymperis K., Stathopoulos V., Coelho L., Rebola A., Vrachopoulos M.G. (2020). Investigating the performance of a thermal energy storage unit with paraffin as phase change material, targeting buildings’ cooling needs: An experimental approach. Int. J. Thermofluids.

[96] Tyagi V.V., Buddhi D., Kothari R., Tyagi S.K. (2012). Phase change material (PCM) based thermal management system for cool energy storage application in building: An experimental study. Energy Build..

[97] McKenna P., Turner W.J.N., Finn D.P. (2021). Thermal energy storage using phase change material: Analysis of partial tank charging and discharging on system performance in a building cooling application. Appl. Therm. Eng..

[98] Zhang S., Yan Y., Cheng Z., Wang F. (2024). Charging and discharging in thermal energy storage unit with fin-stone hybrid structure for enhancing heat transfer of phase change materials. Int. J. Heat Mass Transf..

[99] Nair A.M., Wilson C., Kamkari B., Hodge S., Jun Huang M., Griffiths P., Hewitt N.J. (2024). Energy and exergy analysis of a multipass macro-encapsulated phase change material/expanded graphite composite thermal energy storage for domestic hot water applications. Energy Convers. Manag. X.

[100] Ji C., Waqas H., Liu D., Kang C., Muhammad T. (2024). Melting performance improvement of phase change materials with thermal energy storage unit using nanoparticles. Case Stud. Therm. Eng..

[101] Izadi M., Alshuraiaan B., Sheremet M., Galal A.M. (2023). Melting process of a phase change material using single and double elliptic charger applied in thermal storage for building application: A numerical study on effect of position and aspect ratio. J. Energy Storage.

[102] Jabbar W.K., Alshara A.K., Allawy A.S. (2024). Numerical analysis of a solar thermal energy storage tank filled with phase change material under three fin arrangements. Int. J. Thermofluids.

[103] Taghavi M., Poikelispää M., Agrawal V., Syrjälä S., Joronen T. (2023). Numerical investigation of a plate heat exchanger thermal energy storage system with phase change material. J. Energy Storage.

[104] Rashid F.L., Khalaf A.F., Alizadeh A.A., Al-Obaidi M.A., Salahshour S., Chan C.K. (2024). Numerical investigation of the effect of the number of fins on the phase-change material melting inside a shell-and-tube cylindrical thermal energy storage. Case Stud. Therm. Eng..

[105] Hassan H.M.A., Hansen M.P.R., Dallaire J., Larsen M.A.B., Kieseritzky E., Alexandersen J., Veje C., Lund I. (2023). Performance analysis of a stand-alone thermal energy storage system based on CSM plates filled with phase change material. Energy Build..

[106] Karaağaç M.O. (2024). Performance evaluation of nano-enhanced phase change materials for thermal energy storage: An experimental study. Case Stud. Therm. Eng..

[107] Raheem A.I., Pesteei S.M., Yaqob B.N. (2024). Experimental study on thermocline behavior and management in phase change material-enhanced thermal energy storage: Implications for charging and discharging cycles. Energy Convers. Manag. X.

[108] Reboli T., Ferrando M., Traverso A., Chiu J.N.W. (2023). Thermal energy storage based on cold phase change materials: Discharge phase assessment. J. Energy Storage.

[109] Hathal M.M., Al-Jadir T., Al-Sheikh F., Edan M.S., Haider M.J., Rsool R.A., Haider A.J., Badawy T., Al Jubori A.M. (2023). Thermal performance characterization of a thermal energy storage tank with various phase change materials. Int. J. Thermofluids.

[110] Suresh C., Awasthi A., Kumar B., Lee H., Jeon Y. (2026). Comprehensive review on heat pump systems integrated with phase change material-based thermal energy storage for sustainable building heating. Renew. Sustain. Energy Rev..

[111] Ismail M.M., Dincer I., Bicer Y., Saghir M.Z. (2024). Assessment of a solar-powered trigeneration plant integrated with thermal energy storage using phase change materials. Process Saf. Environ. Prot..

[112] Jin X., You S. (2023). Experimental investigation of the solar latent heat thermal energy storage system integrated with salt hydrate phase-change materials. Energy Rep..

[113] Bertaggia N., Reibert D., Knuth P., Zontar D., Brecher C. (2024). Improving the Energy Efficiency of Machine Tools using a Thermal Storage Unit based on a Phase Change Material. Procedia CIRP.

[114] Khader M.A., Ghavami M., Al-Zaili J., Sayma A.I. (2024). Residential Micro-CHP system with integrated phase change material thermal energy storage. Energy.

[115] Kazaz O., Karimi N., Kumar S., Falcone G., Paul M.C. (2024). Thermally enhanced nanocomposite phase change material slurry for solar-thermal energy storage. J. Energy Storage.

[116] Rinaldi G., Lazaro A., Delgado M., Marin J.M., Verda V. (2024). Use of a low-cost phase change material emulsion in de-centralized thermal energy storage for district heating network enlargement. Energy.

[117] Suresh C., Awasthi A., Lee D., Jeon Y. (2024). Energy and economic evaluation of combined sensible-latent thermal energy storage system with various volume fractions of phase change material. Alex. Eng. J..

[118] Odoi-Yorke F., Opoku R., Davis F., Obeng G.Y. (2023). Optimisation of thermal energy storage systems incorporated with phase change materials for sustainable energy supply: A systematic review. Energy Rep..

[119] Menezes M.V.P., Othman A.-H., Tancabel J., Aute V.C. (2026). An approach for fast and accurate simulation of phase change material based thermal energy storage in buildings. J. Energy Storage.

[120] Wang J., Yu Z., Jia X., Pan B., Liu X. (2026). A comprehensive investigation and multi-objective optimization on enhancing thermal energy storage efficiency of phase change material embedded in partially filled metal foam. J. Energy Storage.

